# Waste-based triboelectric nanogenerators for sustainable energy harvesting: a comprehensive review of materials, mechanisms, and energy applications

**DOI:** 10.1039/d6ra05670a

**Published:** 2026-10-01

**Authors:** Barshan Dev, Mitu Gharami, Khadiza Newaz, Md Ashikur Rahman

**Affiliations:** a Department of Textile Engineering, BGMEA University of Fashion and Technology Dhaka-1230 Bangladesh barshan.butex@gmail.com mitu.duet@gmail.com; b Department of Textile Machinery Design and Maintenance, Bangladesh University of Textiles Dhaka-1208 Bangladesh khadizanewaz2801@gmail.com; c Department of Apparel Manufacturing and Technology, BGMEA University of Fashion and Technology Dhaka-1230 Bangladesh ashikur.buft020@gmail.com

## Abstract

The rapid accumulation of waste materials and the rising demand for clean energy have encouraged the development of sustainable technologies capable of converting waste into functional power-generating devices. Waste-based triboelectric nanogenerators (TENGs) represent a sustainable and emerging class of energy-harvesting devices that convert mechanical energy into electricity while utilizing abundant waste-derived materials. This review summarizes recent advances in the use of agricultural residues, fruit and vegetable peels, textile waste, paper waste, plastics, and industrial by-products as triboelectric materials. This discussion emphasizes the prospective benefits of materials obtained from waste, such as economical feedstocks, extensive accessibility, varied surface chemistry, and possibilities for waste valorization. The fundamental operating mechanism of TENGs based on contact electrification and electrostatic induction is briefly outlined, along with key material engineering strategies, such as surface modification, nanostructuring, and composite formation to enhance performance. The review also presents recent developments and applications of waste-based TENGs in wearable electronics, self-powered sensors, environmental monitoring, healthcare, and IoT systems that offer a viable pathway for combining waste valorization with renewable energy harvesting, supporting the development of sustainable and eco-friendly next-generation energy technologies.

## Abstract 2

Waste-derived resources are engineered into sustainable TENGs through surface modification and nanostructuring for efficient, eco-friendly energy generation.

## Graphical abstract

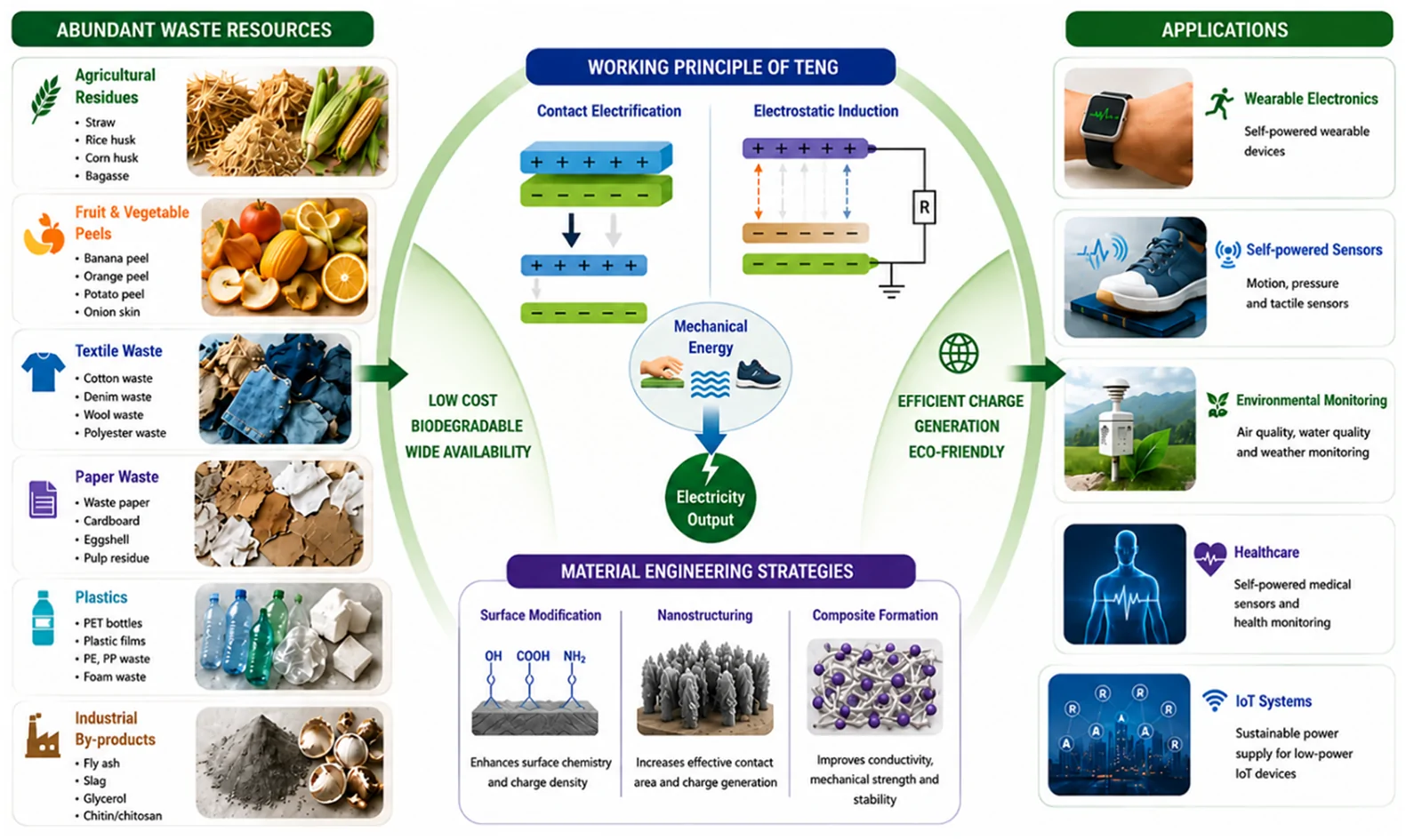

## 1. Introduction

The swift expansion of industrialization, urbanization, and demographic development has resulted in a significant rise in resource utilization and waste production. Concurrently, there has been a notable escalation in the utilization of resource-intensive materials, coupled with a commensurate increase in the indiscriminate discharge of waste into the environment.^1,2^ The global challenge of waste management has emerged as one of the most pressing environmental concerns of our era. The prevailing linear model of “take, make, dispose” has engendered overflowing landfills, polluted waterways, and deleterious impacts on ecosystems. The categorization of what constitutes “waste” is inherently subjective, influenced by temporal, spatial, economic, legal, and individual preferential factors. As articulated by the European Union (EU), waste is defined as “any substance or object which the holder discards or intends or is required to discard”.^2–5^

Waste materials emanate from diverse sources, including manufacturing processes, industrial activities, and municipal solid waste (MSW). MSW encompasses waste from durable goods, nondurable goods, containers, packaging, food scraps, yard trimmings, and miscellaneous inorganic waste originating from residential, commercial, and industrial sources. Moreover, the escalating volume of plastic waste poses a strain on MSW management systems by diminishing available landfill space and giving rise to potential toxic emissions during incineration, including the release of lead and cadmium in ash.^6,7^ The burgeoning issue of electronic waste (e-waste) represents a rapidly growing global concern, posing significant threats to both the environment and human health due to the presence of various toxic substances. The generation of waste carries substantial environmental repercussions, with a direct correlation observed between the rates of solid waste production and the emission of greenhouse gases. Waste management encompasses the treatment of wastewater, sewage, as well as chemical and biochemical processing. Unregulated disposal practices contribute to significant contamination by heavy metals in water, soil, and plant life. Open burning of waste materials further exacerbates environmental concerns by emitting pollutants such as carbon monoxide (CO), carbon dioxide (CO_2_), sulfur dioxide (SO_2_), nitrogen oxides (NO), and particulate matter (PM10), adversely affecting the atmosphere. Individuals engaged in waste picking activities within open dump sites face substantial health risks. Additionally, the discharge of solid waste into water bodies exacerbates the global issue of marine litter, further intensifying environmental pollution.^8–10^

Effective waste management systems can address the significant problem of extensive waste generation and mitigate associated environmental impacts. Addressing this crisis necessitates a paradigm shift towards sustainable practices, and at the forefront of this transformation is the pivotal role of recycling. Recycling is a beacon of hope in the waste management crisis, providing various 21st-century benefits. Key among them is resource conservation recycling materials like paper, glass, plastic, and metals minimizes the demand for virgin resources, helping preserve forests and reduce the environmental impact of resource extraction. Moreover, recycling saves energy by utilizing manufacturing processes that typically require less energy than those for virgin materials. The utilization of microelectronic devices, such as electronic watches and sports bracelets, is experiencing a concurrent increase. While the power consumption of an individual electronic device remains minimal, the collective power usage of these devices, propelled by their popularity and widespread adoption, cannot be disregarded.^10–12^

The upsurge in power generation associated with the heightened usage of microelectronic devices, such as electronic watches and sports bracelets, contributes to an increase in greenhouse gas emissions derived from power generation fuels. This phenomenon significantly impacts the equilibrium of the global carbon cycle.^13^ The exclusive dependence on conventional energy sources proves insufficient to meet the present energy demands, underscoring the imperative for the development and implementation of renewable energy.^14^ In response to the escalating energy crisis and environmental pollution, there is a universal quest for suitable new energy sources.^15,16^ The principle of energy conservation posits that energy is neither created nor annihilated; rather, it undergoes transformation from one form to another. The surrounding environment encompasses various forms of energy, with mechanical energy being the most extensive and occupying a significant portion.^17,18^

In this context, the advent of triboelectric nanogenerators (TENGs) addressed the predicament of energy scarcity. Operating on the principles of triboelectrification and electrostatic coupling effects, TENGs convert surrounding low-frequency energy into electrical energy, thereby achieving energy-to-electricity conversion.^19^ Waste TENGs represent a pioneering advancement in sustainable energy technology, situated at the intersection of waste valorization, mechanical energy harvesting, and self-powered sensing. Their primary innovation lies not only in electricity generation but also in tackling two critical global challenges waste accumulation and energy deficits by providing a dual solution that transforms discarded materials into functional components or harnesses kinetic energy typically squandered in waste streams and management processes. Waste TENGs exemplify the pragmatic implementation of circular economy principles.^16,20–23^ Consequently, TENGs find applications in diverse self-powered systems and triboelectric devices. This technology represents a groundbreaking and environmentally friendly approach to energy harvesting, emerging as a central focus in nanotechnology research. Noteworthy attributes include high output performance, stability, and sensitivity, enabling their incorporation into various structures to provide low-maintenance, long-running, and self-powered energy for microelectronic systems.^24–26^ In recent years, numerous review articles have been published on TENGs, mainly focusing on triboelectric mechanisms, device architecture, nanomaterials, and self-powered sensing technologies. A further knowledge gap concerns the relationship between material and device engineering strategies, triboelectric mechanisms and potential opportunities and challenges in the implementation of waste-based TENGs. However, comprehensive reviews specifically dedicated to waste-derived triboelectric materials, their mechanism, modification strategies to enhance TENG output, implementation challenges and diverse application remain comparatively limited.

This study seeks not only to aggregate documented works on waste-derived TENG but also to create a framework encompassing materials, mechanisms, performance, and applications for comprehending the evolution of waste-based TENG. The assessment methodically categorizes waste-derived triboelectric materials based on their triboelectric polarity, chemical composition, and source of waste with the investigation of the essential mechanisms behind charge generation. This study also elucidates how surface chemistry, morphology, dielectric characteristics, and material combinations affect triboelectric efficacy and appraise modification and hybridization techniques that aimed at enhancing charge generation, retention, durability, and electrical output. Additionally, this review highlights various applications in diverse fields, conducts a comparative analysis of multiple waste-based TENGs, identifies outstanding scientific, manufacturing, environmental, and standardization hurdles, and proposes a pragmatic outlook for future research directions that need to be tackled for effective implementation.

## 2. Waste-derived materials for TENG development

TENGs have gained attention as an innovative energy harvesting technology that can transform low frequency mechanical energy from surrounding environments into practical electrical energy through the combined effects of triboelectric charging and electrostatic induction.^27^ Unlike traditional power generation systems, TENGs excel at capturing irregular and low-intensity mechanical inputs such as human movement, wind, vibrations, and biomechanical activities, making them ideal for powering low power electronic devices, wearable sensors, and self-sustaining systems.^28^ A significant benefit of TENG technology is its exceptional energy conversion efficiency at low operating frequencies, where conventional electromagnetic generators face considerable performance challenges.^29^ Furthermore, the straightforward device design, lightweight nature, and scalable production methods of TENGs facilitate cost-effective manufacturing, enhancing their suitability for large area and flexible energy-harvesting applications.^30^

### 2.1. Waste TENG prepared with animal-based waste

#### 2.1.1. Eggshell membrane

Eggshell membranes (ESMs), composed of collagen types I, V, and X, present a promising biodegradable material due to their abundant functional groups (–COOH, –NH_2_) and natural fibrous structure. These properties make ESMs highly suitable for material design applications. Consequently, efficient EM-based electrode material has been developed for supercapacitor use. Additionally, the nanofibrous architecture and diverse protein composition of ESMs enable functionalities such as piezoelectricity, triboelectricity, capacitance, and humidity sensing.^31,32^ To construct piezoelectric triboelectric nanogenerators (P-TENGs), raw, heated, and carbonized ESMs were combined with ePTFE, which demonstrated efficient triboelectric energy harvesting because of the strong electron-accepting tendency of ePTFE and the electron-donating characteristics of ESM. The P-TENG was employed to monitor wrist motions by affixing it to the wrist, functioning as a wearable electronic device that generates electrical power while also facilitating the observation of human activities and health.^33^

The ostrich ESM-TENG, which produced an output voltage of up to 300 V, is shown in Fig. 1 along with its structure and working mechanism. The device can be used as a smart sensor in a variety of wearable electronic gadgets because its output power of 0.18 mW is sufficient to power 250 red light-emitting diodes (LEDs).^31^ The documented efficacy of eggshell-membrane-based TENGs suggests that the triboelectric properties are closely linked to the membrane's surface functional groups. Variations in electrical output among the documented devices can be ascribed to the selection of counter triboelectric layer, surface morphology, and device arrangement, rather than solely the waste material.^31–33^

**Fig. 1:**
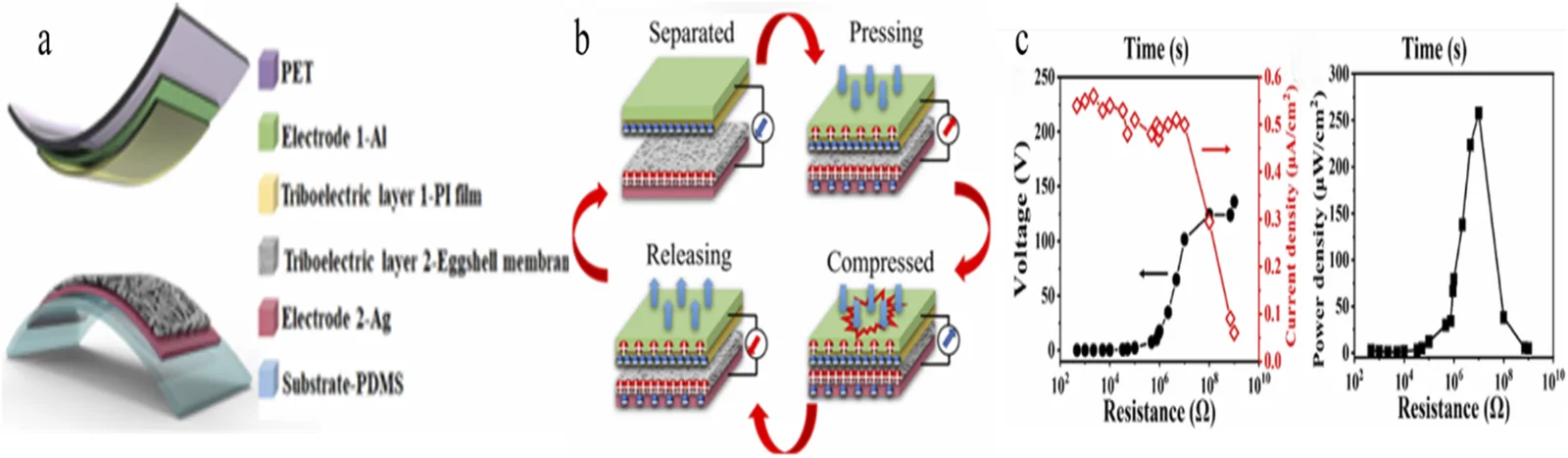
(a) Schematic structure of the ESM-TENG device, (b) working mechanism of ESM-TENG, (c) output voltage and power density of the ostrich ESM-TENG in relation to external load resistance.^31^ Adapted from ref. 31 with permission from American Chemical Society,^31^ copyright 2023.

#### 2.1.2. Chicken skin

Chicken skin (CS), a byproduct of the poultry industry, functions as a viable material in the manufacture of TENGs owing to its intrinsic biocompatibility and biodegradability. The collagen protein in CS imparts strength and elasticity, rendering it an essential structural element that improves the device's functionality. Furthermore, collagen functions as an electropositive material, facilitating effective energy production. CS typically consists of approximately 55% water, 35% connective tissue, and 5–10% fat, making it a readily available and cost-effective resource. Incorporating domestic waste such as CS into TENGs (CS-TENG) supports circular economy principles by converting waste into valuable energy-harvesting devices, thereby mitigating environmental impact and promoting sustainability.^34,35^

Fig. 2 depicts the CS-TENG, which was able to energize as many as 55 commercial LEDs, operate a calculator, and track human physiological movements. The electric output of the 9 cm^2^ CS-TENG may attain 123 V and 20 µA, respectively, signifying a substantial progression into green, battery free energy alternatives.^34^ The efficacy of chicken-skin-derived TENGs illustrates that protein-laden waste can yield substantial triboelectric performance due to its chemically reactive surface and polar functional groups. Nonetheless, a direct comparison with alternative protein-derived wastes must take into account variations in surface treatment, thickness, contact area, counter material, and testing conditions, as these factors might significantly affect the observed results.^34,35^

**Fig. 2:**
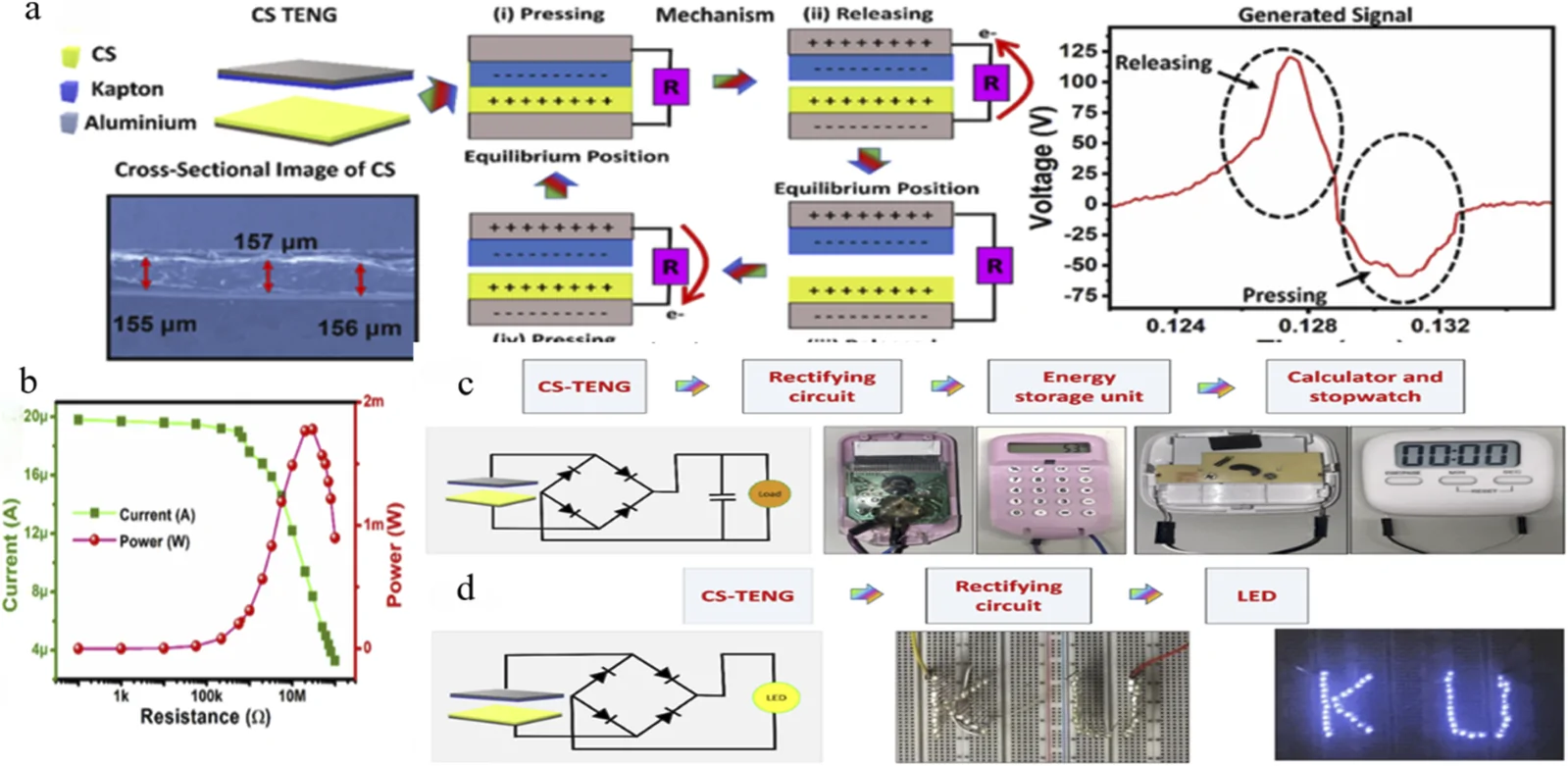
(a) Diagrammatic illustration of the construction and operation of the triboelectric nanogenerator based on chicken skin (CS-TENG), (b) output current and power across different resistances, (c) and (d) the circuit diagram illustrates the use of the CS-TENG with electronic devices such as a calculator, stopwatch, and LED.^34^ Adapted from ref. 34 with permission from Springer Nature,^34^ copyright 2023.

#### 2.1.3. Human hair

Human hair demonstrates a significant triboelectric effect, frequently regarded as an inconvenience in everyday life, resulting from its intricate combination of keratinized proteins, lipids, and moisture.^36^ Human skin and hair exhibit significant positive tribo-polarity, leading to numerous studies being done on TENGs. Notwithstanding these encouraging attributes, the incorporation of human hair into functioning TENG devices has yet to be realized due to difficulties in processing it into appropriate formats. Research has investigated the utilization of human hair in the fabrication of electrodes for Li-ion batteries and supercapacitors, underscoring its potential in energy-related technologies.^37,38^

In Fig. 3(a) bio-TENG constructed from discarded human hair and waste rubber band films, with complimentary micro and surface structures, demonstrated an exceptionally high electrical output and achieved a maximum open-circuit voltage (*V*_oc_) of 7.24 kV and a short-circuit current (*I*_sc_) of 196.44 µA, yielding an outstanding output power density of 28 459.43 µW cm^−2^ for a device area of 5 × 10 cm^2^.^36^ The voltage charging characteristics of 10, 22, 33, and 47 µF capacitors in Fig. 3(b) reveal that the electricity produced by the natural rubber and activated carbon from human hair based TENG could be efficiently captured and stored in commercially available capacitors.^39^ In Fig. 3(c) showed the representation of the manufacturing of AC-HH powder, the creation of PVDF@AC-HH composite TENG, and its numerous uses in gesture detection, energy harvesting, and photo responsive self-powered micro/nanoelectronics systems.^38^ Therefore, changes in functional groups, surface roughness, moisture content, and electron-donating or electron-accepting properties may contribute to discrepancies in charge generation when compared to other biological wastes.^36,38^

**Fig. 3:**
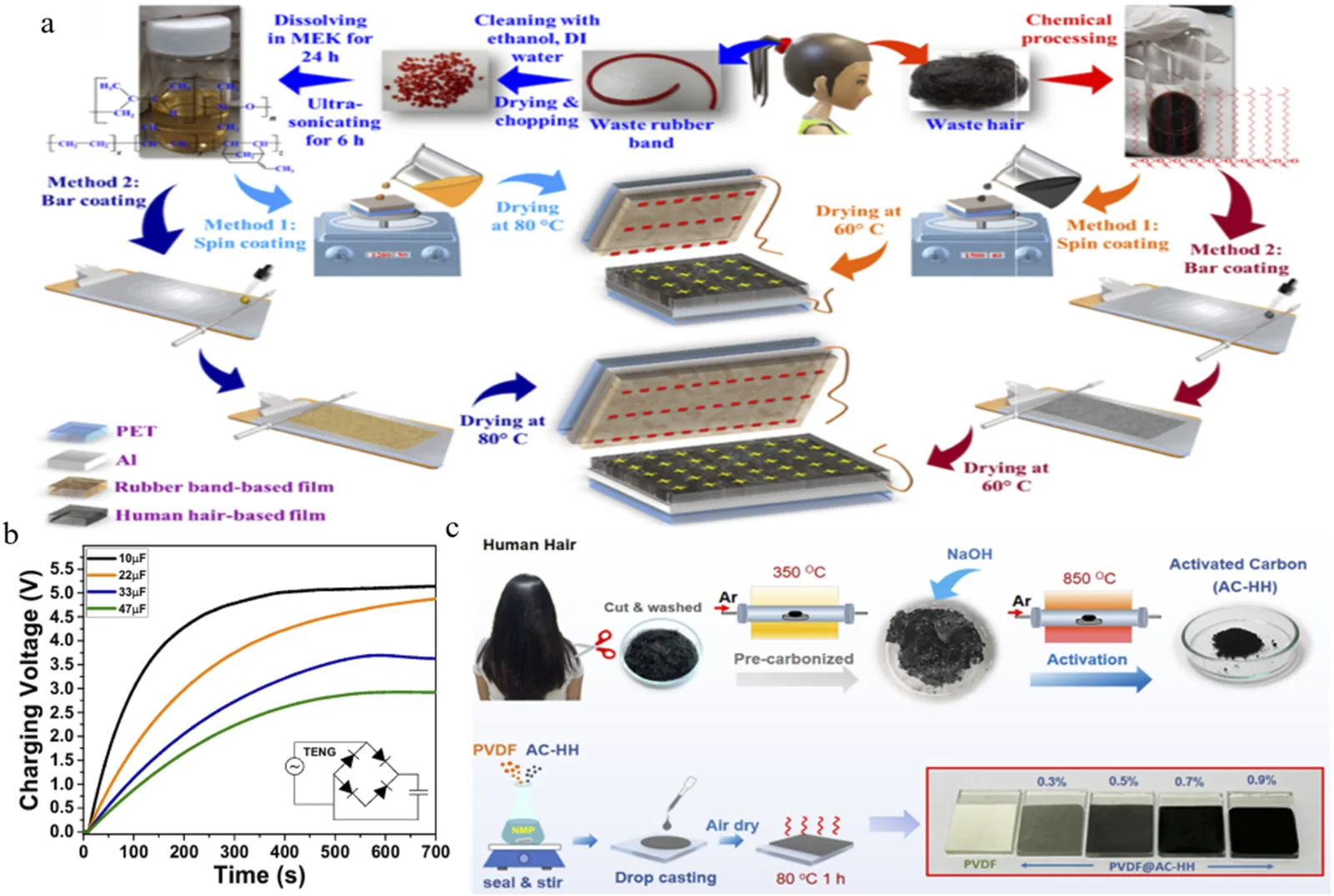
Schematic fabrication process and working mechanism of the human-hair-based triboelectric nanogenerator, showing the preparation of the hair-derived triboelectric layer, electrode integration, and electrical generation under mechanical contact and separation. (a) Fabrication flow of human hair/rubber band-based green TENG. Adapted from ref. 36 with permission from Elsevier,^36^ copyright 2025. (b) Capacitor charging profiles driven by the fabricated TENG. Adapted from ref. 39 with permission from MDPI,^39^ copyright 2022. (c) AC-HH powder preparation and PVDF@AC-HH composite-based TENG fabrication. Adapted from ref. 38 with permission from Elsevier,^38^ copyright 2024.

#### 2.1.4. Fish organ

Gelatin is a natural biomacromolecule extensively employed throughout various industries, including food, medicine, cosmetics, and photography. It has gained prominence due to its beneficial attributes, including cost-effectiveness, enhanced biocompatibility, and efficient degradability. Fish gelatin has supplanted mammalian gelatin, traditionally derived from byproducts of the meat and leather industries, owing to cultural and health considerations. Fish gelatin is an excellent option for triboelectrification due to its elevated content of amino acid residues containing electron donor groups and its exceptional ability to relinquish electrons during friction.^40,41^

In Fig. 4(a) and (b) green fish gelatin-based triboelectric nanogenerator (FG-TENG) was created utilizing leaf-inspired microstructures, with lotus-leaf-like pyramidal surfaces yielding the most significant enhancement in output. The resultant device exhibits up to 5.8 times voltage and 3.8 times current improvement as shown in Fig. 4(d) and (e). Fig. 4(c) showed their non-structured, single-structured, and double-structured layer. The device also exhibited exceptional durability exceeding 10 000 cycles, and considerable potential for sustainable energy harvesting and sensing applications. Fig. 4(c) will be removed from this line.^42^ The presence of polar functional groups in the protein structure, which can take part in interfacial charge transfer, is responsible for the performance of fish-gelatin-based TENGs. Further evidence that surface microstructure and triboelectric coupling have a significant role in influencing charge density and electrical performance comes from the reported variations in output.^40–42^ Various properties of animal-based waste TENG are given in Table 1.

**Fig. 4:**
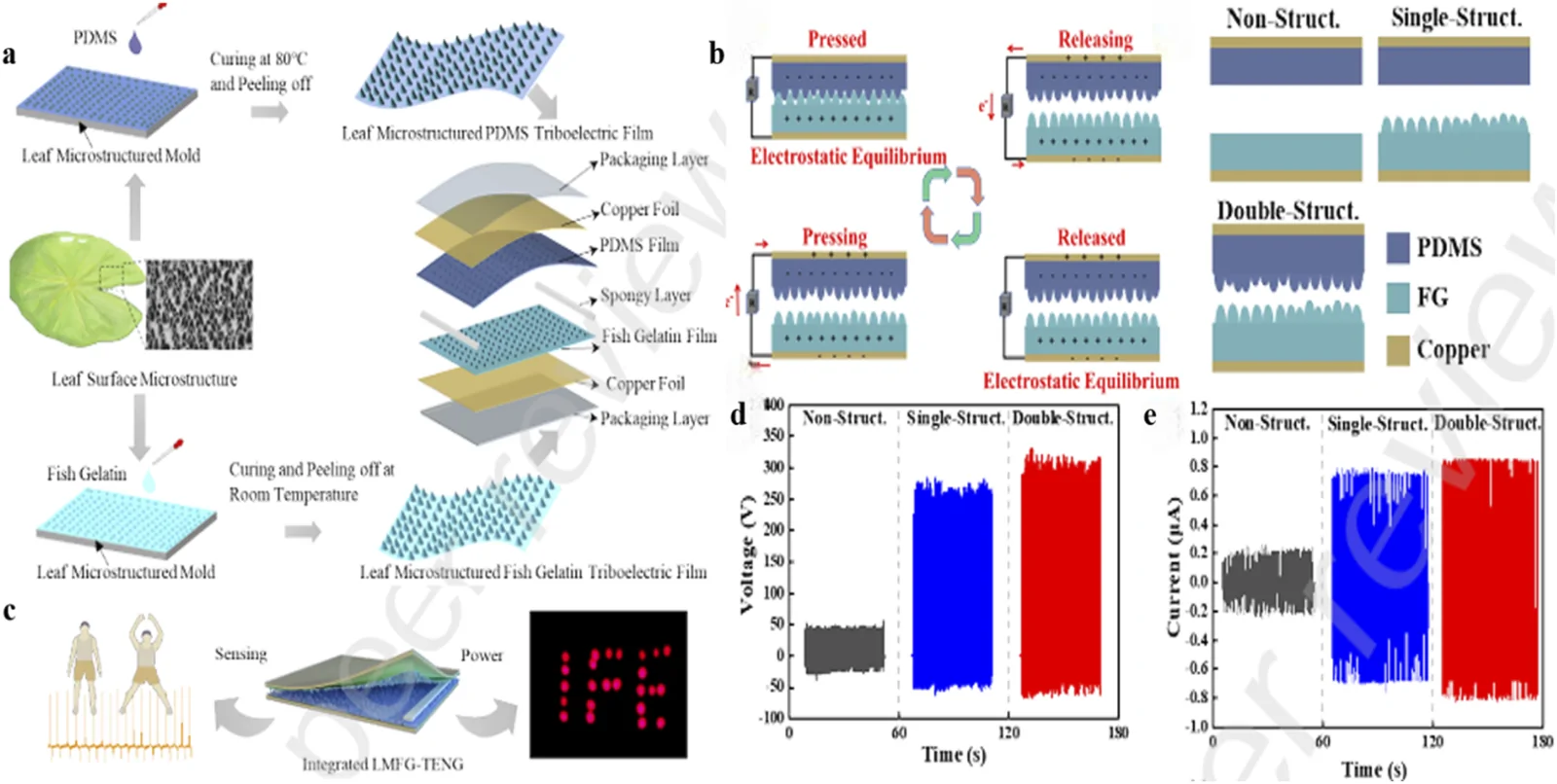
(a) and (c) Development and conceptual representation of a leaf microstructure-inspired fish gelatin-based triboelectric nanogenerator (LMFG-TENG) including autonomous sensing and energy production capabilities, (b) illustration of non-structured, single-structured, and double-structured layers, (d) and (e) voltage and current. Reproduced from ref. 42 with permission from Elsevier,^42^ copyright 2023.

**Table 1:** Summary of numerous animal-based waste derived materials used in TENGs

Waste material	Surface treatment	TENG mode	Polarity of waste-derived layer	Current (µA)	Voltage (V)	Power density/charge accumulation	Key findings	Application area	Ref.
ESMs	PDMS	Contact-separation	Positive	0.6	300	270 µW cm^−2^	High output voltage with improved charge density	Smart sensor	31
ESMs	PTFE	Contact-separation	Positive	15.95	∼150	∼20 nC	High energy harvesting efficiency and motion sensing capability	Wearable devices for biomedical	33
CS	—	Contact-separation	Positive	20	123	0.20 µW cm^−2^	Supports sustainability and enables battery-less wearable devices	Body motion detection	34
Human hair	Rubber bands, methyl ethyl ketone and NaOH	Contact-separation	Positive	196.44	7240	28 459.43 µW cm^−2^	Light weight, flexible, and highly efficient	Self-powered modern health care	36
Human hair	Cryogenic treatment	Contact-separation	Positive	∼4	∼300	∼327 µW m^−2^	Improved charge generation and storage capabilities	LED array illumination, alarm activation, and wireless communication	37
Human hair	Carbonization and NaOH	Contact-separation	Positive	13	200	0.92 W m^−2^	Enhances overall TENG performance	Motion detection and photo response	38
Human hair	Natural rubber	Contact-separation	Positive	6.9	89.6	242 µW m^−2^	Efficient mechanical-to-electrical energy conversion	Motion sensing	39
Fish scale	PDMS, Cu	Contact-separation, sliding mode	Positive	∼0.8	∼320	—	Demonstrates excellent durability and maintains stable electrical output after 10 000 cyclic tests	Electrical powering and physical motion monitoring	42
Shrimp shell	Natural rubber	Contact-separation	Positive	—	∼106	—	Demonstrates significantly enhanced electrical output	TENG for energy harvesting	43
Fish bladder film	—	Contact-separation	Positive	7.3	106	—	Shows linear and stable response to mechanical and environmental stimuli	Wearable electronics, position monitoring	44

#### 2.1.5. Spider silk

Recombinant spider silk (RSSP-TENG) proteins were modified to form biofunctional TENG apparatus with programmable triboelectric characteristics, where silk proteins facilitate controllable electron affinity and superior mechanical durability, which enhances charge transfer during contact electrification. Furthermore, micro-structured surfaces were produced using a water-lithography approach, which increases surface roughness and contact area and boosts electrical output. The resulting spider-silk-based TENG showed great stability and a high-power output around 145 V where *V*_oc_, demonstrating its potential for self-powered sensors, wearable electronics, and biomedical devices.^45^

Overall, the triboelectric performance of animal-based waste-derived materials is governed by their chemical composition, triboelectric polarity, surface chemistry, dielectric properties, and surface morphology rather than by waste type alone. Protein-rich materials such as eggshell membrane, chicken skin, human hair, and fish gelatin contain functional groups from collagen or keratin, which influence their electron-donating/accepting ability and interfacial charge generation. Differences in functional-group composition, surface roughness, moisture content, dielectric properties, and interaction with the counter triboelectric layer therefore leads to substantial variations in electrical output.

### 2.2. Waste TENG prepared with plastic waste

#### 2.2.1. Waste plastic bags (WPB)

Humans generate vast quantities of plastic and electronic waste, contributing to environmental pollution, particularly in water sources. The escalating use of fossil fuels further exacerbates ecological harm. TENG presents an affordable and eco-friendly solution to mitigate these challenges. TENGs can be created by recycling plastic, converting biomechanical energy into power. Their versatile production methods enable the incorporation of unique features. Growing interest in stretchable electronics for various applications, including energy devices, sensors, and wearable electronics, has emerged. Current batteries face challenges in meeting weight, lifespan, and power delivery criteria. TENGs, with their simple design, diverse materials, low cost, and exceptional power output, offer a sustainable alternative for portable devices.^46–50^ This growing problem adversely affects drainage systems, marine ecosystems, and the environment at large, is chiefly caused by the rising output of plastic goods and insufficient recycling methods. The increasing popularity of low-power electronic devices has heightened concerns about energy consumption. The concurrent resolution of these difficulties through the repurpose of discarded plastic bags as frictional materials in TENGs can transform mechanical energy produced by routine movements into a consistent and stable electrical output.^51^ These devices utilize materials including silicone rubber and polymers derived from discarded plastic bags. A standard TENG is made up of two layers that are intended to generate electrical charges through friction. These layers can be combined with plastic bags to efficiently record mechanical motion. The captured energy may then be stored in capacitors or batteries or directly employed to power electronic devices.^51^ Nylon and polyvinyl chloride (PVC) based TENG (PA-PVC-TENG), nylon and polyethylene (PE) based TENG (PA-PE-TENG), and PVC and PE based TENG (PVC-PE TENG) were fabricated utilizing the most common PA, PVC, and PE plastic films as the triboelectric layer. The PA-PVC-TENG exhibited exceptional output performance, attaining an *V*_oc_ of 35.7 V, a *I*_sc_ of 5.85 µA, and a peak output power density of 152.6 mW m^−2^.^52^ The details properties of several plastic waste-based materials used in TENGs are given in Table 2.

**Table 2:** Summary of several plastic waste-based materials used in TENGs

Waste materials	Integrated materials/surface treatment	TENG mode	Polarity of waste-derived layer	Current (µA)	Voltage (V)	Power density	Key findings	Application area	Ref.
PA-PVC, PA-PE, and PVC-PE	—	Contact-separation	Positive	5.85, 4.71, and 2.45	35.7, 27.3, and 14.8	152.6, 98.2, and 22.5 mW m^−2^	PA-PVC-TENG demonstrates better performance	LED	51
WPB	PTFE	Contact-separation	—	34.1	251	1.57 µW cm^−2^	Enables strain detection without external power source	Power devices and functional sensor devices	52
PVC	Nylon	Contact-separation	Negative	3.1	31	11.9 mW m^−2^	Enables self-powered Morse code communication system	Morse code generator, LED	53
PVC waste pipe	Nylon, ZnO	Contact-separation	—	12.4	392	726.3 µW cm^−2^	Improved performance by adding ZnO nanoparticles to enhance piezoelectric properties	Wearable energy harvesting, automatic switches, and invisible sensors	54
Wood-plastic composite	Teflon	Contact-separation	—	30	31	107 µW m^−2^	Can power around 80 LEDs and portable electronic devices	Energy-harvesting devices	55
Polystyrene	PTFE	Contact-separation	Positive	52	250	4.05 W m^−2^	Good stability up to 20 000 cycles and 180 days	IoT and smart sensing systems	56
Polystyrene	Al	Contact-separation	Positive	67	690	28 W m^−2^	Spray-coated PS showed best performance	Charging capacitors, powering calculator, running remote controlled car	57
Polystyrene	Tetrahydrofuran (THF) and *N*,*N*-dimethylformamide (DMF)	Contact-separation	—	12	200	77 mW m^−2^	Performance comparable to advanced piezoelectric fluoropolymers	Environmental remediation	58
PET bottles	Trifluoroacetic acid and dichloromethane	Contact-separation	Positive	9.4	67.7	5 W m^−2^	Soft, flexible, porous, and structurally durable material	Environmental remediation	59
PET bottles	PBAT, Cu	Contact-separation	Negative	∼25	∼250	16 µW cm^−2^	Harvest sufficient energy to illuminate up to 100 LEDs	Robotics, automobile industries, tire motion monitoring in rovers and aerospace electronics	60
Plastic water bottle	Graphite	Contact-separation	Positive	101	83.88	26.54 µW cm^−2^	Converts biomechanical energy into electricity	Self-powered portable electronics	61
PTFE plastic waste bottle	Al	Contact-separation	Positive	45	1400	300 µW cm^−2^	Good charge retention and fast response	Charging capacitors, powering calculator	62
Polybags, food wrappers, grocery bags and plastic bottles	Al, PU foam	Contact-separation	Positive	289 nA	44	3.2 mW m^−2^	Effectively harvests biomechanical energy to power	Dynamic force sensor and various household application	63
PTFE (waste plastic coffee cup)	Super P conductive carbon black, polyvinylidene fluoride, *N*-methyl-2-pyrrolidone	Contact-separation	Positive	2.5	—	0.61 1.57 mW cm^−2^	Effectively supports sustainable, self-powered, and IoT-enabled technologies	Self-powered sensors and internet of things systems	64

#### 2.2.2. PVC

The demand for stretchable and flexible electronics has increased, especially for applications in energy harvesting, biomedical devices, smart sensors, actuators, luminescent systems, and both wearable and attachable electronics, such as electronic and ionic skins. The widespread implementation of these technologies is constrained by the limited lifespan, weight, and power density of conventional batteries and capacitors, which hinder a reliable and sustainable power source. TENGs have garnered interest as a feasible alternative energy source owing to their simple design, material versatility, low production costs, ease of fabrication, and remarkable output performance, particularly under low-frequency mechanical movements.^53,65^

Recent developments have shown that plasticized PVC gel can be successfully used to create a single-layer, stretchy, transparent, and attachable TENG. This PVC gel based stretchy TENG demonstrates exceptional performance in biomechanical energy harvesting and tactile sensing applications. Without sacrificing mechanical or optical qualities, the PVC gel's stretchability and transparency allow for close conformal contact with intricate surfaces, such as human skin. When the stretchable TENG is interfaced with different materials including nylon, polyethylene terephthalate (PET), and polyethylene naphthalate (PEN), it efficiently produces electrical output by contact electrification. It is especially well-suited for wearable and self-powered sensing systems since it can communicate directly with the human finger.^53,65,66^ This advancement makes PVC gel based stretchable TENGs adaptable parts for the upcoming generation of wearable electronics, providing improved user comfort and self-sufficiency in power generation. Their capacity to capture biomechanical energy from routine motions and transform it into electrical signals that can be used creates opportunities for human–machine interfaces, continuous health monitoring, and other applications that need dependable, adaptable, and lightweight power sources. PVC based TENGs are an appealing way to get around the drawbacks of traditional batteries in flexible and stretchable electronic devices because of their mechanical flexibility, optical transparency, and effective energy conversion.^65,66^

Recycled PVC from waste pipes as shown in Fig. 5(a) which was combined with 0.5 wt% ZnO nanoparticles and electrospun into nanofibrous mats for triboelectric generation of energy. Nylon-6 nanofibers surpassed metallic electrodes, such as copper, due to their advantageous triboelectric polarity and mechanical flexibility. The improved PVC/ZnO and Nylon-6 device attained a power density of 726.3 µW cm^−2^ and maintained around 90% output stability during prolonged execution, with the gathered energy being adequate to power 192 green and 50 red LEDs. Its potential for self-powered sensing applications highlighted by its sensitive finger-force detection and efficient conversion of biomechanical movements as shown in Fig. 5(b). In Fig. 5(c) illustrates a hypothetical schematic of the internal network of adipate-plasticized PVC gel, whereby PVC strands are physically linked to adipate plasticizer molecules *via* secondary interactions rather than covalent bonds. The mechanical characteristics of PVC gels were meticulously examined using uniaxial tensile testing, with PVC-to-plasticizer (DMA & DOA) ratios ranging from 1 : 1 to 1 : 5. Young's modulus ranged from 0.19 to 0.79 MPa at a ratio of 1 : 3, indicating an ideal balance between mechanical strength and softness as shown in Fig. 5(d). Long-term stability is essential since the plasticizer is not chemically linked to the PVC structure. The aging process of PVC films being plasticized with DMA and DOA during a 30 days period is shown in Fig. 5(e). The migration and evaporation of the plasticizer caused the film containing DMA to significantly shrink and lose weight. The PVC film containing DOA, on the other hand, kept its original dimensions and look, suggesting improved plasticizer retention and long-term structural stability.^66^

**Fig. 5:**
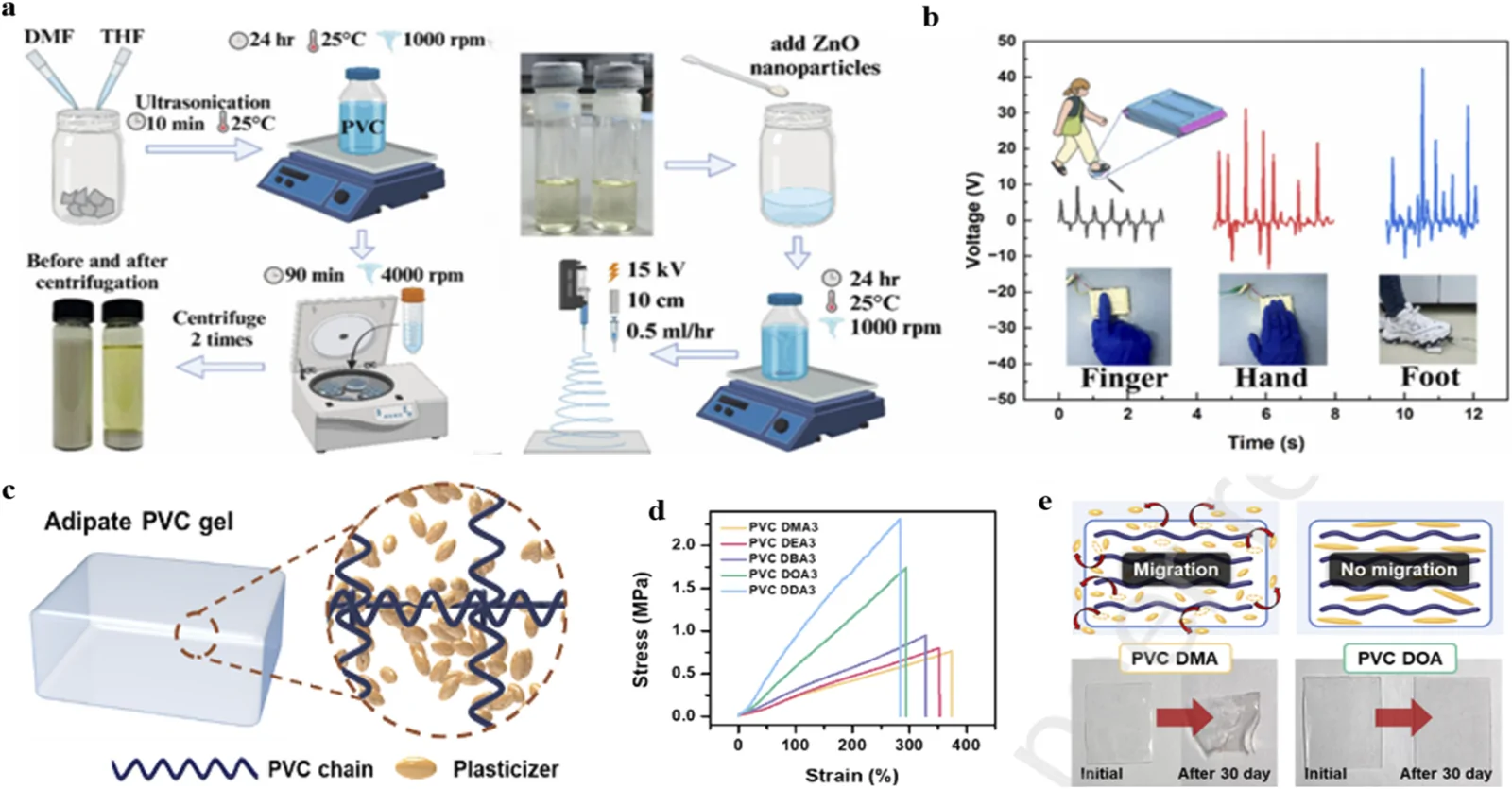
(a) Schematic illustration and electrospinning process for recycling PVC/ZnO NFs, (b) self-powered sensing applications highlighted by its sensitive finger-force detection. Reproduced from ref. 54 with permission from Wiley,^54^ copyright 2025. (c) Diagram depicting the adipate-plasticized PVC gel's microstructural arrangement, (d) stress–strain curve of various plasticizer-based PVC gel, (e) long-term dimensional reliability of PVC gels plasticized with DMA and DOA. Reproduced from ref. 66 with permission from Elsevier,^66^ copyright 2023.

#### 2.2.3. Waste polystyrene

Waste polymers like polyurethane foam (PU), expanded polystyrene (PS), polypropylene (PP), PE foam, and melamine formaldehyde foam (MF) have been used to create environmentally friendly, simple, and affordable TENGs without the need for chemical or physical modification.^47,58^ From the garbage packaging scrap, PS-based TENG was developed which demonstrated the best electrical performance that have maximal *V*_oc_ of 690 V, a *I*_sc_ of 67 mA, and a maximum output power density of 28 W m^−2^.^47^ In a separate investigation, PS derived from diverse packaging substance and leftover PTFE tape functioned as triboelectric layers, resulting in a WPS-TENG that demonstrated an output voltage of approximately 200 to 250 V, a peak power density of 4.05 W m^−2^, and a charge density of around 90 µC m^−2^.^56^

#### 2.2.4. PET plastic bottles

According to recent studies, flexible plastic products including drinking straws, bags, and packaging films are overtaking stiff polymers like PET in the packaging business. Despite being widely used, PET does not biodegrade naturally, which causes it to accumulate over time and cause serious environmental damage when disposed of. Furthermore, burning petroleum-based polymers like PET emits toxic pollutants that worsen air pollution, accelerate ozone layer depletion, and worsen global warming.^59,61,67^ Furthermore, another study as shown in Fig. 6(a) exhibited the formation of a three-dimensional PET aerogel using see-through PET bottles. This process involved mixing trifluoroacetic acid, dichloromethane, and PET material to produce the PET solution. In Fig. 6(b) PEI grafting, which partially obscured the pDA particles and changed the surface chemistry, caused a small reduction in fiber surface roughness. Boasting a maximum peak voltage of 67.7 V and a maximum current of 9.4 µA, it has an exceptionally large ability to remove different heavy metals and store energy.^59^

**Fig. 6:**
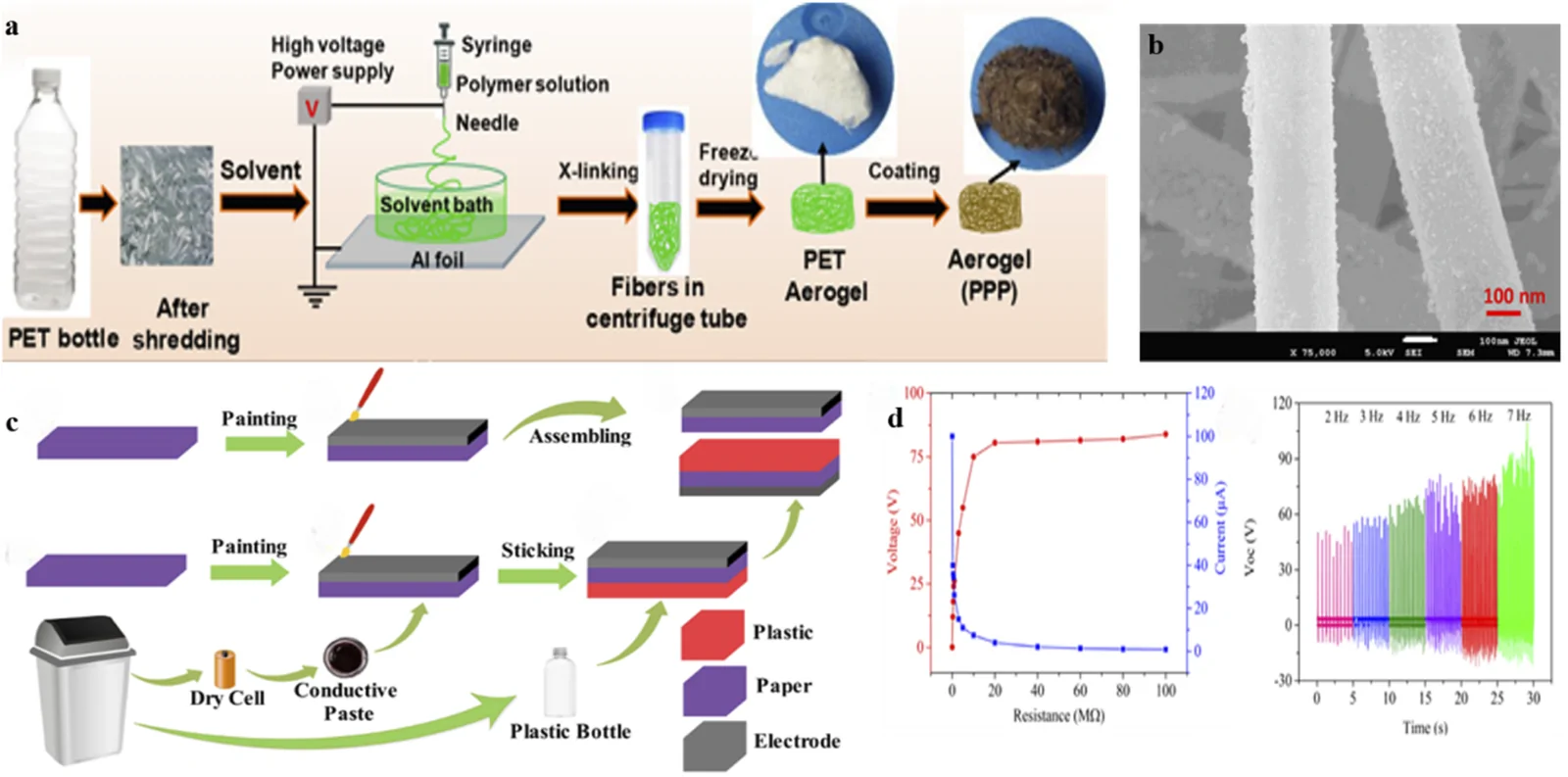
(a) Diagram showing the procedure of the fabrication of a 3D aerogel from discarded PET bottles, (b) FESEM images depicting PET-pDA-PEI. Reproduced from ref. 59 with permission from Elsevier,^59^ copyright 2021. (c) Schematic representing the graphite and plastic based TENG (GP-TENG) fabrication process utilizing paper electrodes and recycled plastic, (d) electrical performance of the GP-TENG demonstrating output current and voltage across varying external load resistances and operating frequencies. Reproduced from ref. 61 with permission from Elsevier,^61^ copyright 2022.

In Fig. 6(c), electrodes were made from recycled paper substrates by using a brush to apply a conductive paste based on graphite that was obtained from waste dry cells. After that, drying was done at room temperature without the need of solvents or specialist equipment. To finish the device's assembly, a plastic sheet made from used bottles was attached to one electrode. The resulting GP-TENG demonstrates its capacity to efficiently power small electronic devices with an *V*_oc_ of 83.88 V, a *I*_sc_ of 101 µA, and a peak power density of 26.54 µW cm^−2^ as shown in Fig. 6(d).^61^

#### 2.2.5. PTFE plastic bottles

The creation of a TENG made from inexpensive, easily accessible materials like graphene oxide, aluminum foil, and PTFE waste bottles is an important innovation. Double-sided sticky tape was used to mold and attach PTFE waste bottles that were gathered from domestic trash to act as the aluminum foil electrode. Graphene oxide, which serves as a negative triboelectric layer and has carefully positioned holes to allow electrical connections, was applied to the underside of the TENG. This method provides a cost-effective and sustainable way to generate power by effectively utilizing non-biodegradable materials to harness the triboelectric effect.^62^ The triboelectric performance of plastic waste-derived materials varies mainly with triboelectric polarity, surface chemistry, dielectric properties, surface morphology, and the nature of the counter triboelectric layer. Materials such as PVC, PS, PET, PE, and PTFE exhibit different electron-donating or electron-accepting tendencies, resulting in different charge-generation capabilities. In particular, the highly electronegative nature of PTFE generally favors negative triboelectric behavior, whereas polymers such as nylon act as positive triboelectric materials. Surface modification, roughness, porosity, and incorporation of fillers further enhance charge generation by increasing effective contact area and interfacial charge density.^48,68^

### 2.3. Waste TENG prepared with plant-based waste

#### 2.3.1. Peanut shell powder

Potentially using peanut shells powder, TENGs provide a sustainable and environmentally benign way to produce electricity. In order to permit mechanical movement, this creative method entails choosing appropriate materials, building a dual-layered device, and incorporating the TENG into a system or application. Electrical energy produced by triboelectric charging can be collected, stored in capacitors, or utilized to power small electronics. Low-power electronics, wearable technology, food processing, education, and agricultural sensors are among potential uses. The design of the device and the caliber of the materials used can affect how successful a TENG employing peanut shell powder is. For economical and ecologically friendly mechanical energy harvesting, biowaste-based, flexible, non-toxic TENGs with excellent performance are advised.^69^

#### 2.3.2. Sunflower husk powder

Recycling sunflower husks, a substantial bio-waste from sunflower seeds, can reduce resource consumption and environmental damage. Sunflower husks are high in cellulose, the most prevalent natural polymer made up of repeated glucose units forming polysaccharides, with a composition of about 48.4% cellulose and 17.0% lignin. Each of these glucose units has three hydroxyl groups, whose oxygen atoms promote electron donation and speed up the triboelectrification process. Sunflower husk powder can be used as a sustainable, biowaste-based material to create TENGs by utilizing these inherent chemical features. These TENGs provide low-cost mechanical energy harvesting solutions that are nontoxic, flexible, and environmentally benign. They may find use in wearable technology, low-power electronics, and environmental sensors. Device design and material quality have a significant impact on the performance of TENGs made from sunflower husk powder, highlighting the significance of optimizing these factors for increased energy conversion efficiency.^70,71^ The details of the plant waste-based materials used in TENGs are given in Table 3.

**Table 3:** Summary of plant waste-based materials used in TENGs

Waste materials	Integrated materials/surface treatment	TENG mode	Polarity of waste-derived layer	Current (µA)	Voltage (V)	Power density	Key findings	Application area	Ref.
Peanut shell powder	Al, Cu	Contact-separation	Positive	104.5	910	12 mW cm^−2^	Powers 150 LEDs and an electronic calculator	Self-powered electronic devices	69
Peanut skin powder	Al, Cu, PET	Contact-separation	Positive	0.2	162	2.2 mW m^−2^	Successfully powers humidity sensor without an external power source	Humidity sensor	72
Sunflower husks powder	Al, Cu, PET	Contact-separation	Positive	28.5	488	1200 µW cm^−2^	Can light up 153 LEDs	Microelectronic devices (*i.e.*, calculator and stopwatch)	70
Coffee waste	Silicone rubber elastomer	Contact-separation	—	2.1	130	60 mW m^−2^	Lightweight, flexible, shape-adaptive, and stretchable	Wearable power and self-powered smart sensors	73
Rice husk	PDMS, PTFE, PET, Cu, SiO_2_	Contact-separation	Positive	14	270	0.84 W m^−2^	Shows lower electron affinity compared to commercial SiO_2_ nanoparticles	LEDs	74
Squandered cotton	PVA	Contact-separation	Positive	50	600	8.45 mW cm^−2^	Successfully harvests energy from human foot movement when placed under the medial arch	Powering portable electronics	75
Wood pulp	Nitro and methyl functionalization	Contact-separation	—	9	8	—	Nitro and methyl functionalization significantly enhance triboelectric performance	Powering small electronic devices	76
Corn husk	Cu film	Contact-separation	Positive	8.67	480	67.05 µW cm^−2^	Demonstrates excellent durability with stable output over 25 211 cycles at 14 Hz	LEDs, digital calculators, and stopwatches	77
Fruit shells: almonds, walnuts, and pistachios	—	Contact-separation	Positive	95	700	416 mW cm^−2^	Pistachio waste shell shows maximum triboelectric performance due to higher cellulose and oxygen content	Wearable and portable devices	78
Rice paper-PVC	—	Contact-separation	—	6	244	37.64 µW cm^−2^	Devices are stable, recyclable, and environmentally friendly	Paper-based electronic, LED	79
Tea leaves	Aluminum plastic bags	Contact-separation	Positive	42.8	792	488.8 mW cm^−2^	Can power 179 green LEDs	Energy harvesting and sensing for office environments	80
Wheat straw	Acrylic and Cu tape	Single electrode	Negative	12	250	40.4 mW cm^−2^	Ability to power more than 60 LEDs	Portable device, environment monitoring, wind speed detection	81
Rose petal	Poly(methyl methacrylate)	Contact-separation	Positive	0.78	30.6	2.72 µW cm^−2^	Demonstrates the potential of using living plant structures for environmental energy harvesting	Powering electronics	82
Aloe vera leaf	PDMS	Contact separation and free standing	Positive	200 nA	1.2	0.22 µW cm^−2^	Demonstrates as a self-powered finger motion sensor for monitoring finger bending angles	Motion sensing and powering wearable electronics	83
Orange peels	PDMS	Contact-separation	—	—	90	∼135 µW cm^−2^	Exhibits strong electrical performance	Wearable electronics and smart home systems	84
Tomato peels	Cu, PET	Contact-separation	Positive	84	150	—	The porous structure of tomato peel significantly improves charge generation and energy conversion efficiency	Wearable devices and portable electronics	85

#### 2.3.3. Waste tea leaves

The production of TENGs from used aluminum plastic bags and spent tea leaves possesses a sustainable method that tackles several environmental issues. In addition to minimizing the depletion of natural resources, this approach drastically cuts environmental pollution and energy usage. By utilizing the special surface characteristics of leftover tea leaves as a functional material, the triboelectric effect in these TENGs allows for the effective conversion of mechanical energy into electrical energy. This waste-derived TENG technology presents a viable way to advance the production of renewable energy by incorporating it into wearable electronics, energy-harvesting surfaces, and self-powered gadgets. These uses could result in environmentally friendly power solutions that make use of cheap, plentiful waste materials, supporting the ideas of the circular economy and lowering dependency on traditional energy sources.^80^

Waste tea leaves and polytetrafluoroethylene (PTFE) film recovered from aluminum plastic bags containing layers of PE and PVC make up the triboelectric pairs as shown in Fig. 7(a–d). A unique TAP-TENG structure in the shape of a honeycomb lantern has been created to effectively harvest mechanical energy from surrounding sources. This novel arrangement detects a variety of human tactile interactions, such as knocking, pressing, and stroking, in addition to utilizing mechanical motions like book turning and drawer pulling. The compact TAP-TENG device could power 179 commercial green LEDs connected in series *via* a full wave bridge rectifier. Furthermore, a dedicated power management circuit has been developed to convert TENG's inherently high-voltage alternating current (AC) output into a stable direct current (DC) output of 3.3 V. This regulated power supply enables the reliable operation of various electronic office devices, such as digital temperature-humidity meters, calculators, and electronic watches.^80^

**Fig. 7:**
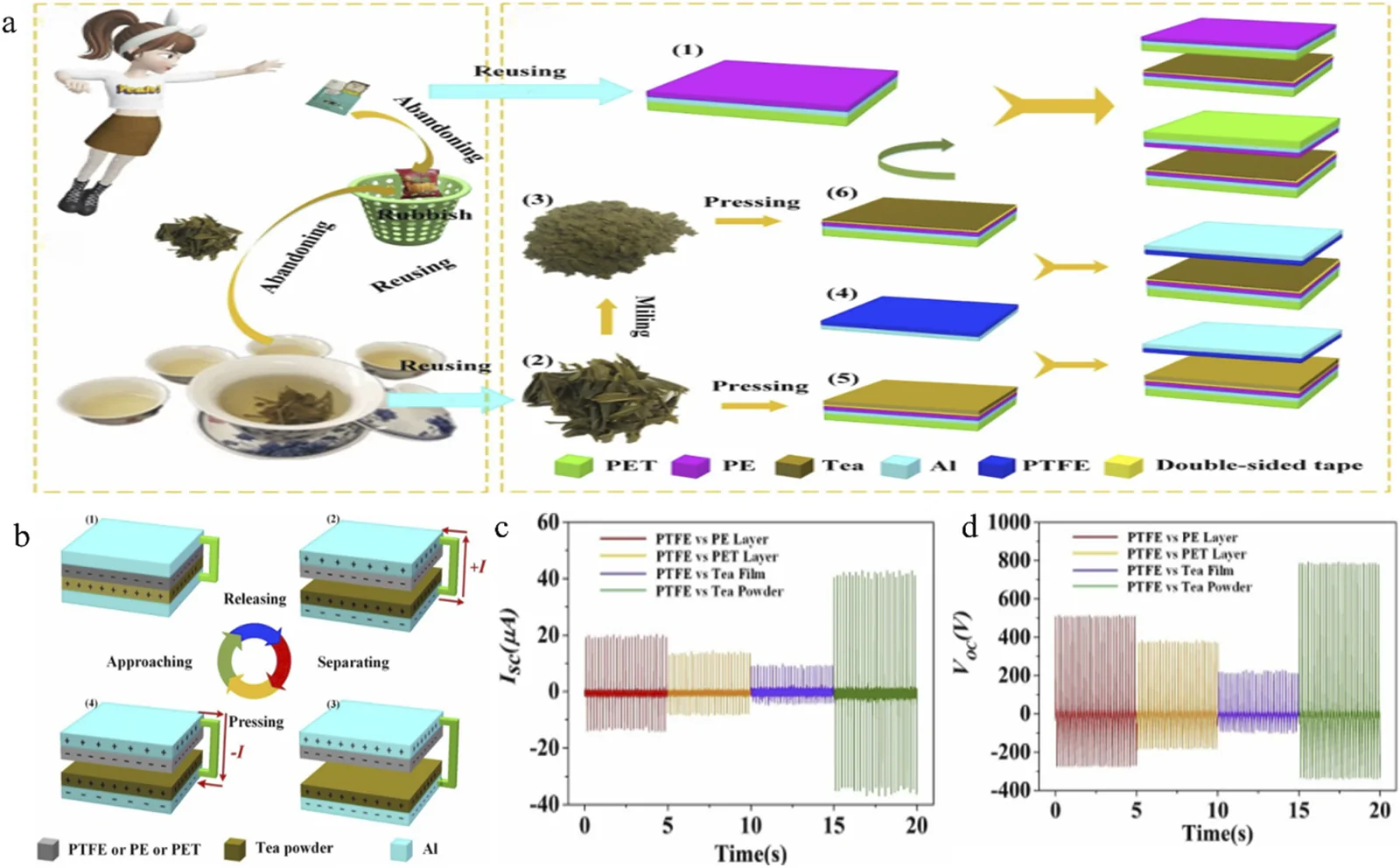
(a) Preparation route of the tea leaf and aluminum plastic triboelectric nanogenerator (TAP-TENG), (b) working method of (TAP-TENG), (c) and (d) current and voltage. Reproduced from ref. 80 with permission from Elsevier,^80^ copyright 2019.

#### 2.3.4. Coffee waste

A solution to waste and environmental problems is provided by the triboelectric nanogenerator, which generates electricity from discarded coffee debris. Over eight million tonnes of waste coffee grounds are produced by the coffee industry each year; these grounds can be used as affordable carbon precursors. A variety of carbon compounds, such as particle-like graphene, activated carbon, mesoporous carbon, carbon nanosheets, carbon nanotubes, and graphene sheet fibers, can be made from leftover coffee grounds.^73^

To create green TENGs for harvesting mechanically, the CG-TENG was created by simply treating discarded coffee grounds. The coffee waste served as a flexible plastic raw material for the conductive electrode as shown in Fig. 8(a) and (b). It can be readily stretched to more than five times its initial length, as given in Fig. 8(c) and (d) which is comparable to silicone rubber's flexibility. This suggests that the electrode component's excellent elasticity and longevity are a result of the combination of silicone rubber and C-coffee waste. The SEM picture in Fig. 8(e) demonstrated that silicone rubber and porous C-coffee ground could be evenly combined at a particular weight percentage.^73^

**Fig. 8:**
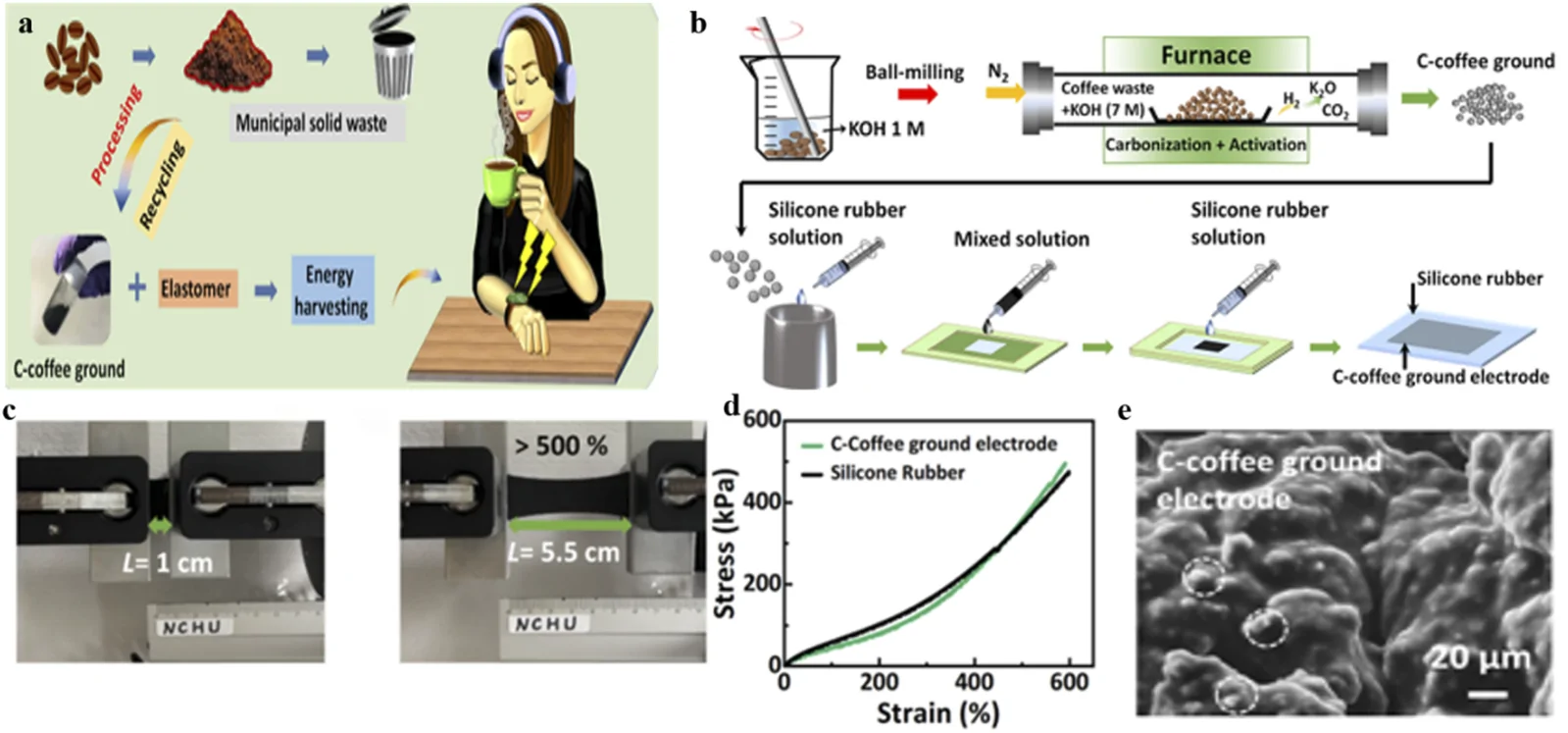
(a) and (b) Diagram depicting the process of coffee ground waste TENG (CG-TENG) utilizing recycled coffee trash, (c) normal state and elongated state of electrode, (d) stress strain curve of C-coffee waste-derived electrode and silicon rubber, (e) SEM photograph of the CG-TENG. Reproduced from ref. 73 with permission from Elsevier,^73^ copyright 2021.

#### 2.3.5. Rubber waste

Utilizing rubber waste, the TENG is an energy harvesting device that uses the triboelectric effect to produce electricity. It is made up of two different kinds of rubber recovered rubber waste and rubber with different electronegativity's. Rubber based TENGs are investigated using a variety of techniques in one investigation. On substrates, NR latex and titanium dioxide (TiO_2_) nanoparticles are cast and cured. The ScCO_2_ foaming technique is used to create silicone rubber/CNFs composites with 0–15 weight percent CNFs combined with dicumyl peroxide (DCP). Another work used high-ammonia natural rubber latex that has been partially de-ammonized, cast, and dried to produce an NR-AgNP TENG.^43,86–88^

#### 2.3.6. Aloe vera

Low cost and environmentally friendly TENG devices can be made by processing aloe vera residues into functional triboelectric layers. In order to produce periodic contact-separation or sliding motions, the fabrication method usually entails careful material selection, the creation of a multilayer device architecture, and the integration of the TENG into mechanically active systems. Electrical output from electrostatic induction and triboelectric charge creation can be collected, stored in capacitors, or used immediately to power low-power electronics.^83,89^

#### 2.3.7. Rice husks

The basic idea is that when materials with different triboelectric qualities come into contact and then separate, a potential difference is created, which produces electricity. The sustainability of this strategy is highlighted by using rice husks, a byproduct of rice milling, as a renewable resource. The production of nanoporous RH_SiO_2__ using centrifugation, washing, thermal annealing, and acid hydrolysis is the focus of a significant investigation. The current density of 5.7 mA m^−2^ was observed from the RH_SiO_2__ TENG, indicating that the porous RH_SiO_2__ film can provide a fast charging and discharging process due to its wide range of pore size distribution.^74^

#### 2.3.8. Rice paper

A groundbreaking study created a biodegradable TENG by triboelectrically combining PVC and rice paper. Transparent conductive ink is applied on PET substrates wrapped with PVC tape to generate the rice paper TENG (RP-TENG), which is renowned for its transparency and flexibility. The RP-TENGs electronic performance was assessed, and the approximate output voltage and short circuit current values were 244 V and 6 µA, respectively.^79^

#### 2.3.9. Coconut husk

A bio-based TENG has been engineered utilizing coconut husk waste as a sustainable tribopositive material. Cellulose from coconut husk is integrated with TiO_2_ nanoparticles to enhance surface roughness and augment charge generation. The incorporation of TiO_2_ amplifies contact electrification and increases the effective surface area, leading to heightened voltage and power output. A lateral sliding mode TENG design was employed to optimize the conversion of mechanical energy into electrical energy. The refined device achieved significant *V*_oc_ and power density, demonstrating its efficacy for low-power, self-powered sensor applications. This study highlights the potential of coconut waste as an economical, eco-friendly material for renewable energy harvesting devices.^90^

#### 2.3.10. Corn husk

Corn husk has recently been investigated as a viable triboelectric material for waste-derived TENGs. Its cellulose-rich composition and roughness on the exterior enable effective electron donation during contact electrification when combined with polymer materials like polystyrene. The device exhibited stable electrical output with a power density of approximately 670.5 mW m^−2^, capable of powering small electronics such as LEDs and calculators. The inherent microstructure of corn husk enhances the contact area between triboelectric layers, thereby improving charge generation efficiency.^91,92^

Plant-based waste-derived TENGs exhibit diverse triboelectric performance depending on their cellulose, lignin, and other functional components, surface chemistry, morphology, dielectric properties, and triboelectric polarity. Cellulose-rich wastes such as sunflower husk, coconut husk, corn husk, rice paper, and peanut shell generally provide abundant hydroxyl groups that can facilitate electron donation and interfacial charge generation, while lignin and other surface constituents modify the dielectric and charge-transfer behavior. Surface roughness, porosity, and microstructure also influence the effective contact area and charge density. In some systems, carbonization or incorporation of fillers such as TiO_2_ enhances conductivity, dielectric properties, surface roughness, or charge trapping, resulting in improved electrical output.^69–71,90^

### 2.4. Waste TENG prepared with medical waste

#### 2.4.1. Surgical face mask

Repurposing medical plastic waste for energy applications has grown in importance in accordance with the ideas of sustainable development and the circular economy. In this study, PP from old surgical face masks that had been UV-C sterilized was used to create a TENG. With a modest tapping force of 3 N, the TENG produced from this waste effectively absorbed low-level mechanical energy and was able to power small electronic devices such as digital thermometer, clock, and calculator. This device demonstrates competitive or even better performance with less input force when compared to previously recorded devices, highlighting its potential for wearable energy harvesting. This approach facilitates the production of value-added energy from medical waste while directly addressing the problem of plastic pollution brought on by the epidemic.^93,94^

#### 2.4.2. X-ray film

From the various types of medical waste discarded X-ray sheets and silicone are utilized as frictional layers for manufacturing TENG, with the system designed for sustainability by including easily recyclable components and ecologically benign materials. The suggested TENG generated an output voltage of 201 V, a current of 62.8 mA, an instantaneous power output of 1.39 W m^−2^ and was capable of simultaneously powering 240 red LED. Moreover, it could supply electricity to electronic equipment, including a calculator, digital watch, hygrometer, and thermometer, using rechargeable capacitors.^95,96^

#### 2.4.3. Ointment tubes

Diverse medical waste is integrated into TENG technology to yield exceptional performance. The findings facilitate the reduction of medical waste while producing renewable energy, in accordance with many sustainable development goals (SDGs). Discarded ointment tubes, whether composed of polymer or metal, offer a unique advantage as they do not necessitate supplementary electrode attachment. The epoxy resin covering functions as one triboelectric surface, while the aluminum base functions as the electrode, thereby streamlining the TENG fabrication process. The resulting metallic ointment tube-based TENG generates a maximum output voltage of approximately 405 V and a current of around 82 µA, yielding a power density of 658 mW m^−2^.^97^ The overview of numerous medical and pharmaceutical waste-based materials used in TENGs are given in Table 4.

**Table 4:** Summary of different medical and pharmaceutical waste-based materials used in TENGs

Waste materials	Integrated materials/surface treatment	TENG mode	Polarity of waste-derived layer	Current (µA)	Voltage (V)	Power density	Key findings	Application area	Ref.
Medical waste X-ray film	Silicone	Contact-separation	Positive	62.8	201	1.39 W m^−2^	Capable of directly powering 240 red LEDs	Self-powered devices, sensors, and smart buildings	95
Medical ointment tubes	Al, Cu, Kapton, PET, PMMA, FEP, and silicone	Contact-separation	Positive	82	405	658 mW m^−2^	Powers 480 LEDs and portable electronic devices through an energy management system	Portable electronic devices	97
COVID-19 clinical waste (nitrile gloves and face masks)	—	Contact-separation	Negative	4.8	50.7	6.39 µW cm^−2^	Convert mechanical energy into electrical energy efficiently	Smart sensors	98
Clinical PP	—	Contact-separation	—	4	50	1.8	Can power 15 serially connected LEDs	TENG as energy harvester	99
Expired pharmaceutical drugs	Cu, PET	Contact-separation	Positive	53	561	163	Harvests enough energy to illuminate up to 169 commercial LEDs	TENG as energy harvester	100
Silane bottle (PP)	Silicone	Contact-separation	—	105	508	8.78 µW m^−2^	Can power 420 red LEDs and portable electronics	Health monitoring, tactile sensing, humidity sensing, and energy-harvesting devices	101
Surgical face mask	Mylar sheets, Cu	Contact-separation	Positive	0.29 mA	200	71.16 8.78 mW m^−2^	Low-cost, flexible, and fabricated without complex processing techniques	Calculator, digital thermometer, and LCD clock	93
Medical mask wastes	PEDOT : PSS, Cu, Al	Sliding	—	60	0.8	7.1 mW m^−2^	Demonstrates flexibility, comfort, and reliable performance for wearable applications	Wearable electronics	94
Medical wastes (rubber gloves, paper pill boxes, X-ray films)	Al	Contact-separation	Positive	3.6	180	65 mW m^−2^	Demonstrates effective energy harvesting performance under 3 Hz frequency and 150 N external force	Thermometers, calculators, watches	96

#### 2.4.4. Saline bottle sheets

Another study explores the use of sheets from medical waste saline bottles (SB) as triboelectric materials for the development of energy-harvesting and multi-sensing systems. High power densities were demonstrated by TENGs built in both vertical contact-separation and single-electrode formats, enabling them to light LEDs, portable devices, and health-monitoring systems including humidity and sleep sensors. Compared to other medical plastics, saline bottles provide a safer and more effective option for TENG manufacturing because of their minimal patient involvement, direct utilization without the requirement for polymer blending, and significant waste volume. SB based TENGs are among the best waste-derived systems due to their reliable performance and steady electrical output. The focus of this work is on a scalable and legally compliant way to turn medical plastic waste into useful energy and sensing technology.^102^

#### 2.4.5. COVID-19 clinical waste

The spikes in plastic pollution, aggravated by the global impact of the COVID-19 pandemic, has brought the globe to the edge of an imminent trash crisis. Global plastic pollution levels have dramatically increased due to the extensive usage of required personal safety equipment (PPE), such as masks, gloves, and PPE kits, as well as flaws in waste management systems. An investigation demonstrates the potential of employing COVID-19 clinical waste, like masks and gloves, to produce cheap TENG for energy harvesting and sensing. This ingenious application of recycled clinical waste materials not only confronts the critical issue of plastic pollution but also illustrates the possibilities for sustainable technological solutions.^98,103^ A piece of three-layered mask and nitrile gloves were placed inside the 3D structure as the top negative and bottom positive triboelectric materials with copper and aluminum as corresponding electrodes (MG-CS TENG) that was capable to use touch sensor for IoT cloud-supported sanitizer dispenser and the maximum voltage, current, and power density obtained from the device are 50.7 V, 4.8 µA, and 6.39 µW cm^−2^, respectively and the harvested energy was sufficient to power small-scale electronic devices like digital tally counters, wristwatches, lumex displays, and series connected 25 LEDs. A three-layered mask and nitrile gloves were incorporated into the 3D structure as the top negative and bottom positive triboelectric materials, with MG-CS TENG. This configuration enabled the use of a touch sensor for an IoT cloud-supported sanitizer dispenser. The device achieved maximum voltage, current, and power density of 50.7 V, 4.8 µA, and 6.39 µW cm^−2^, respectively, and the energy generated was adequate for running small-scale electronic devices such as digital tally devices, wristwatches, lumex displays, and a series of 25 interconnected LEDs.^98^

#### 2.4.6. Expired pharmaceutical drugs

Pharmaceuticals are essential for the prevention and treatment of various diseases in humans and animals. These therapeutic agents, synthesized for both humans and animals, contain active chemicals and may result in adverse side effects if not disposed of properly after expiration. The disposal of expired medications is critical, as improper disposal can cause environmental harm, contaminating groundwater and soil. Effective disposal methods for expired medications are highly sought after. Recently, various waste materials have been utilized as electrodes, leveraging the triboelectric phenomenon to generate clean energy through TENG.^100,104^

Medical waste-derived materials demonstrate promising potential for TENG applications, with PP surgical masks, X-ray films, ointment tubes, saline bottle sheets, and COVID-19 clinical waste successfully functioning as triboelectric materials. Among the reported systems, X-ray film/silicone and ointment-tube-based TENGs exhibited relatively high electrical outputs, while saline-bottle-based TENGs demonstrated promising and stable performance for energy harvesting and sensing. The performance differences can be attributed to variations in material type, triboelectric polarity, surface characteristics, device structure, and counter triboelectric material.^93–95,102^

### 2.5. Waste TENG prepared with textile waste

#### 2.5.1. Recycled textile waste

Numerous leftover textile wastes are repurposed for the fabrication of single electrode mode TENG devices, with wool, silk, jute, and cotton serving as the positive triboelectric surface, while Kevlar, polypropylene, and rubber function as the negative triboelectric surface. These devices transform mechanical energy from human movement, such as stretching, running, or athletic activities, into electrical energy *via* triboelectrification and electrostatic induction. Utilizing textile waste in the fabrication of TENGs fosters a circular economy by encouraging recycling, reuse, and minimizing environmental pollution. The knitted textile TENG created in the study produced electrical outputs appropriate for self-powered sensing applications in advanced sports and wearable electronics.^105^

#### 2.5.2. Hemline nylon fabric

The pnG-TENG device employed nylon fabric as the positive triboelectric material and PVC heat transfer vinyl as the negative layer, configured in alternating strips to augment the effective contact area. This freestanding triboelectric-layer mode design enhances charge generation compared to traditional grating structures that incorporate air gaps. The developed pnG-TENG produced an RMS *V*_oc_ of approximately 136 V and a maximum power density of 38.8 mW m^−2^, illustrating its potential for wearable energy harvesting and electronic textiles.^106^

Textile-waste-based TENGs show a clear trend in which triboelectric polarity and material pairing strongly influence charge generation. Natural protein- and cellulose-based textiles such as wool, silk, jute, and cotton generally exhibit tribopositive behavior, while synthetic materials such as polypropylene, Kevlar, rubber, and PVC tend to act as tribonegative counterparts. In addition, increasing effective contact area through textile structures and device configurations can improve triboelectric performance.^105,106^

### 2.6. Waste TENG prepared with domestic waste

#### 2.6.1. Household

Discarded plastic bottles as shown in Fig. 9(a)–(c), fragments were efficiently employed as a structural foundation for a TENG device assembled from commonplace household items. The opposing triboelectric layer was fabricated from assorted waste materials, including plastic bottles, food packaging, grocery bags, and polybags.^63^

**Fig. 9:**
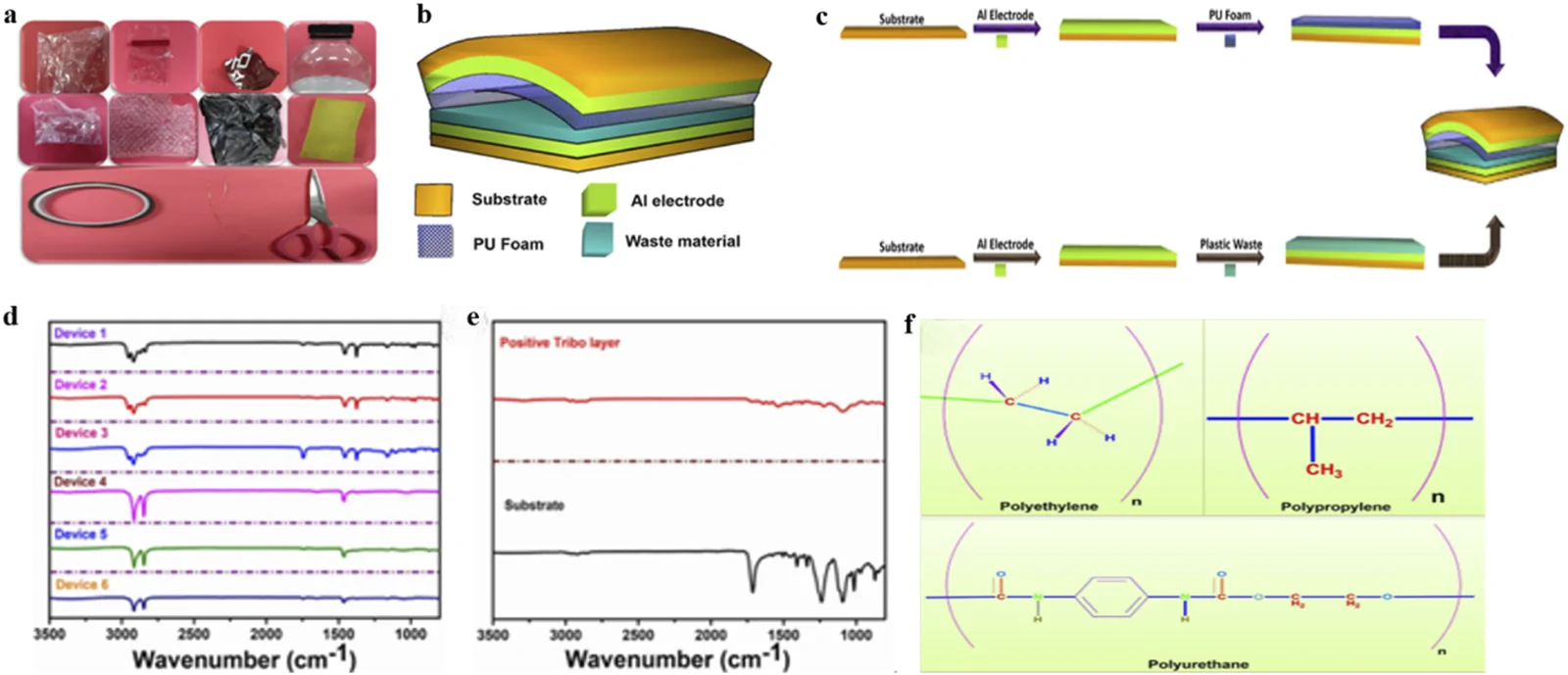
(a) Numerous types of household waste, (b) and (c) diagrammatic representation of the H-TENG production process, (d)–(f) FTIR and chemical structure of the materials. Reproduced from ref. 63 with permission from Elsevier,^63^ copyright 2018.

The complementary triboelectric layer was crafted from a repurposed kitchen sponge, while the electrode was constructed from standard cooking-grade aluminum foil. To measure electrical outputs, copper wiring salvaged from an obsolete compact fluorescent light transformer was utilized and connected to the aluminum electrode. The FTIR and chemical analysis helped to identify the material characteristics as shown in Fig. 9(d)–(f).^63^

#### 2.6.2. Milk carton

The buildup of household garbage, including milk cartons, presents problems with pollution and waste creation. Practical answers to these problems include reusing garbage while acknowledging the possibility of waste material exploitation. The structure of a milk carton, which is made of PE, aluminum (Al), and paperboard, is ideal for creating a TENG because it closely combines an Al conductive barrier with a PE insulating surface. Milk cartons are used as the triboelectric material in this novel six-layer TENG, with the layers acting as the back electrode and dielectric layer.^107^ A self-powered sensor operating wirelessly, utilizing TENG technology, produces electrical energy from mechanical vibrations or motions. In another study, the arc-shaped TENG (AS-TENG) was introduced, a device that integrates an elastic arc-shaped beam and milk carton sheets. TENGs function by converting mechanical energy into electrical energy through the triboelectric effect and electrostatic induction.^108^ The overview of the domestic waste and miscellaneous waste based TENG are given in Table 5.

**Table 5:** Summary of numerous domestic waste and miscellaneous waste-based TENGs

Waste materials	Surface treatment	TENG mode	Polarity of waste-derived layers	Current (µA)	Voltage (V)	Power density/charge accumulation	Key findings	Application area	Ref.
Milk	PTFE	Contact-separation	Positive	93	392	583.7 µW cm^−2^	Can directly power up to 82 blue LEDs (3.4 V)	Food quality detection and powering small electronic devices	109
Cigarette filter-PVC	—	Contact-separation	Positive	0.86	42.8	63.2 µW m^−2^	Generates about 3 V under real-life vibration conditions	Environmental remediation, energy harvesting	110
Aseptic carton packages	Al, Cu	Contact-separation	—	∼400 nA	220	—	Successfully charges a 10 µF capacitor up to 3.5 V in less than 10 minutes	Environmentally friendly destination/solution for food packaging	111
Waste milk cartons	Cu, Kapton, PU foam, acrylic, silicone	Contact-separation	Positive	9.6 mA	9	—	Enhance charge density resulted in efficient energy harvesting performance	TENG for energy harvesting	107
Waste milk cartons	Beryllium bronze, acrylic, Cu	Contact-separation	Positive	40	600	—	Structural parameters significantly improved device performance	Real-time environmental monitoring	108
Cigarette butts	Branched polyethyleneimine	Contact-separation	Positive	11	274	7.54 W m^−2^	Successfully powers small electronic devices such as LED switches	Human motion monitoring, tactile sensing, and UV-shielding	112

#### 2.6.3. Salt

Salt TENG is made from sodium chloride powder in one sheet and PTFE film in another sheet acts as a self-sufficient humidity detector. This salt TENG device can attain an aggregate power of 403.3 µW, with an inner resistance of 30 MΩ and can accomplish an output voltage of 198 V and a *I*_sc_ of 6.66 µA, respectively. The response of salt TENG under varying relative humidity conditions reveals that when relative humidity increases, the electrical output also grows.^113^

Overall, domestic-waste-based TENGs demonstrate that commonly discarded household materials can be directly repurposed as structural, triboelectric, dielectric, or electrode components. Plastic bottles, food packaging, grocery bags, polybags, kitchen sponges, and milk cartons can be integrated with suitable counter materials to generate electrical output. The performance is mainly influenced by the material composition, triboelectric polarity, surface characteristics, dielectric properties, and device configuration. Milk cartons are particularly interesting because their multilayer structure combines PE, aluminum, and paperboard, allowing different functional roles within a single waste-derived materials.^63,108,113^

### 2.7. Waste TENG prepared with miscellaneous waste

#### 2.7.1. Aseptic cartoon package

The fabrication of most TENGs necessitates the utilization of costly materials such as gold and platinum, employing expensive methodologies. These devices' functions are based on the principle of contact electrification and are increasingly acknowledged as effective instruments in energy harvesting technologies. Aseptic carton packages (ACPs) comprise laminated materials, primarily paperboard, PE, and aluminum. A recent study developed a TENG utilizing ACP and oxidized LDPE (LDPEox) as dielectric materials.^111^

#### 2.7.2. Carbon-coated paper wipes

Electrostatic induction and the triboelectric effect are used by TENGs to transform mechanical energy into electrical energy. Because carbon-coated paper is electrically conductive and allows electrons to flow more easily, it can be used in TENGs. Through flow-induced vibration, TENGs with copper electrodes covered in acrylic sheets generate output signals. *N*-Methyl-2-pyrrolidone (NMP), polyvinylidene fluoride (PVDF), super P conductive carbon black, and triboelectric materials from used plastic coffee cups are all included into a flexible, affordable TENG produced from waste plastic and carbon-coated paper wipes. By creating a charge imbalance, the friction between the coated paper and another substance in TENGs harvests mechanical energy.^64^

#### 2.7.3. Cigarette filter

With over 6 trillion cigarettes smoked in 2012 and over 9 trillion predicted by 2025, cigarette filters (CFs) represent a serious environmental risk. Through microplastic pollution, CFs, which are made of the non-biodegradable plastic cellulose acetate (CA), affect both terrestrial and marine environments. Recycling CFs is essential for protecting the environment and producing new, useful materials.^112,114^ The study's objectives were to collect, clean, and dissolve old CFs and make a thick ink using ethanol, DMF, branched polyethyleneimine (bPEI), and plasticizer triacetin (TA). It was cast and oven-treated to produce a thick CA/bPEI/TA film for TENG.^111^

Aseptic carton packages contain paperboard, PE, and aluminum, providing a combination of dielectric, triboelectric, and conductive functions within a single waste material. Carbon-coated paper wipes offer enhanced electrical conductivity and charge transport due to the carbon coating, while cigarette filters composed mainly of cellulose acetate can be chemically reprocessed into triboelectric films. These differences demonstrate that material composition, surface functional groups, conductivity, dielectric behavior, and triboelectric polarity collectively influence charge generation and device performance. Therefore, the functional role and performance of miscellaneous waste materials depend not only on their waste origin but also on their inherent chemical and electrical characteristics and subsequent processing.^111^

## 3. Fabrication process of waste-based TENGs

Triboelectrification and electrostatic induction effects are essential to the operation of TENGs. The four main modes of operation of TENG vertical contact separation, linear sliding, single-electrode, and free-standing modes are being explored as the field of nanogenerators develops. These modes require two different triboelectric materials, suitably linked electrodes, and insulation between layers, facilitating dielectric–dielectric or metal–dielectric configurations. The fundamental premise shared by all four modes is that motion inside any triboelectric layer disturbs the electrostatic equilibrium, leading to a potential difference between electrodes. Repeated mechanical actuation in both forward and backward directions induce the triboelectric layer to generate alternating voltage between electrodes, resulting in positive and negative peaks in the TENG output and ultimately providing an AC signal.^115–119^

As shown in Fig. 10(a), triboelectrification occurs when two triboelectric materials or layers come into contact and separate in the contact separation mode of the TENG. Either two distinct dielectric materials or a mix of metallic and dielectric layers may be used in this procedure. This type is noteworthy for its cost-effectiveness, simple design, and easy construction. For powering low-power devices, it is significant for being the first TENG mode to be invented and demonstrated. The ability to be configured as a multi-unit stack gives this TENG mode additional flexibility and improves output performance.^115,116^ In lateral sliding mode of the TENG, charge generation results from the relative sliding to and from movement between TENG layers. With electrodes affixed to the rear of triboelectric layers, the construction is quite similar to that of the contact separation mode; however, as Fig. 10(b) shows, the displacement takes place laterally. Because of its large contact area, the sliding mode compensates for its longer sliding displacement by having a higher charge density, even though it has a lower figure-of-merit than the vertical contact separation mode. Additionally, by adding more grating structures, this mode can improve output performance. Notably, by using cylindrical grating structures, the sliding mode TENG can also function in a rotating manner.^115,117^ The single electrode mode TENG, which is distinguished by its simplicity, is the most fundamental structure among TENGs. However, minimal charge transfer limits its output performance, resulting in less generated voltage and current. The single-electrode mode TENG is quite effective for self-powered applications despite this drawback. This device's capacity to get over the application restrictions seen in contact separation mode and sliding mode TENGs, where contact wires obstruct on both sides, is one of its advantages. Fig. 10(c) shows the basic configuration of a single-electrode mode TENG.^115,117^

**Fig. 10:**
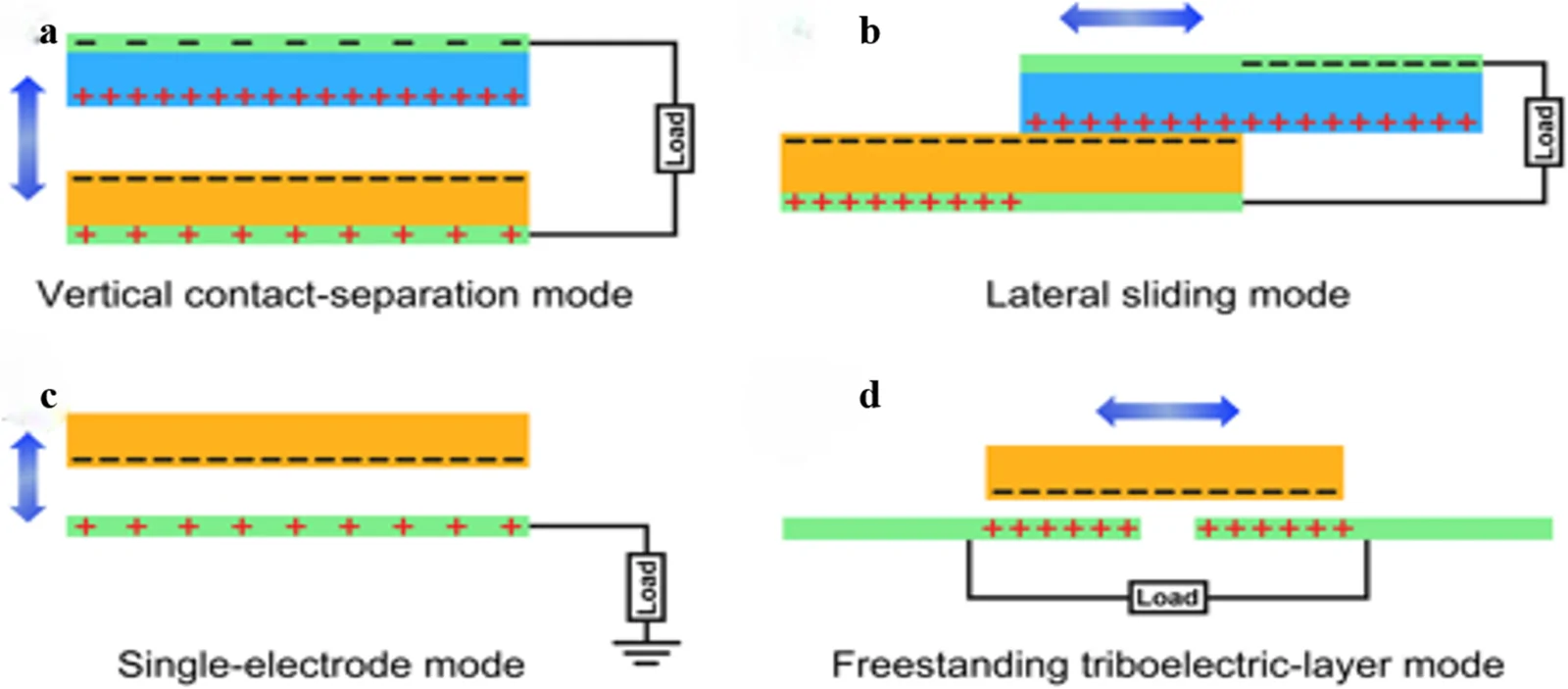
(a)–(d) Diagrammatic display of the four principal functioning phases of TENG. Adapted from ref. 115 with permission from Wiley,^115^ copyright 2020.

A triboelectric layer without an electrode can migrate over two electrodes or triboelectric layers in the free-standing model of TENG, but the electrodes themselves stay stationary. In addition to having a high figure-of-merit, this TENG mode has demonstrated remarkable electrical output and output efficiency. It also provides smooth integration into a variety of real-time applications and ease of manufacture. Fig. 10(d) shows the fundamental setup of a free-standing mode TENG.^115,116^

### 3.1. Fabrication of animal waste based TENG

The fabrication of TENG utilizing animal waste materials involves various methodologies for sustainable energy extraction. The extraction of ESMs for TENG production employs several techniques; while acidic treatment and physical stripping are prevalent, the preferred method is manual removal, followed by deionization and drying. The active layer electrode is created by applying dry ESM to aluminum tape and connecting it to a wire. The second TENG active layer electrode incorporates materials such as calotropis, *Bombax ceiba* tree fruit, tissue paper, dog hair, PTFE, aluminum, and copper.^31–33,120,121^

In a concurrent investigation, ESMs underwent acid treatment, boiling (HESMs), and carbonization (CESMs) to fabricate P-TENGs. This process included the preparation of ePTFE membranes, which were subsequently sectioned into squares and utilized in the assembly of P-TENGs with copper tape serving as electrodes, maintaining a uniform separation distance of 1 cm for optimal contact and separation.^33^ Using an *in situ* polymerization technique, a controllable thickness nanocomposite hydrogel electrolyte was created on biowaste ESMs. The BSA-PDMAA-SiO_2_ cross-linked gel demonstrated high ionic conductivity (8.8 mS cm^−1^) and significantly improved electrochemical performance when compared to pristine ESMs. This hybrid gel eggshell structure shows an effective method for transforming inexpensive natural waste into high-performance solid-state energy storage devices.^32^ TENG was created in a different project using ESMs from ostrich, hen, duck, and goose. Before being incorporated into a stretchy electrode composed of PDMS modified with N_2_ plasma treatment and covered with a 50 nm thick coating of silver. The ostrich EM-based TENG demonstrated exceptional output performance, achieving a peak voltage of 300 V and a maximum power of 0.18 mW, attributable to its elevated surface roughness, dielectric constant, and plentiful functional groups. The constructed device exhibited remarkable durability and was effectively utilized for powering LEDs, wearable electronics, and self-sustaining motion sensing.^31^

Using CS, a waste high in protein, as an environmentally acceptable resource, triboelectric nanogenerators and bio-composite films were produced. The used skins were thoroughly cleaned and processed using simple, economical, and ecologically friendly techniques to create gelatin-based composite films and efficient triboelectric layers.^34,35^ In a groundbreaking study, the wasted CS was collected, properly cleaned with ethanol and deionized water to remove contaminants, and then allowed to air dry at room temperature. After drying, the skin was sliced into 3 × 3 cm^2^ squares and used as the tribopositive layer right away. Aluminum adhesive tape served as the electrode, and Kapton film served as the tribonegative component. With a stacking order of Al/Kapton/spacer/CS/Al and a consistent air gap of 4 mm, the TENG device was built in a vertical contact–separation configuration. To guarantee structural integrity and reliable output performance, PET sheets were employed to mechanically support the entire device, and copper wires were used for electrical connections.^34^ In another investigation fresh CSs were meticulously cleaned to remove fat and impurities, subsequently dried at 45 °C, ground, and subjected to Soxhlet extraction for defatting. Gelatin was extracted *via* a sequence of alkaline, acid, and aqueous treatments, then filtered, concentrated, and freeze-dried to yield gelatin powder. By dissolving 4 g of CS gelatin in distilled water and combining it with different proportions of potato starch (0–10%, w/w), film-forming solutions (FFS) were produced. After being gelatinized at 90 °C, the potato starch was mixed with the gelatin solution and plasticized using 30% (w/w) glycerol. After being poured onto flat Petri dishes, the homogenous FFS was allowed to dry for 72 hours at 45 °C. The films were carefully taken out after drying and kept in desiccators until they were characterized.^35^

Human hair and other waste materials that break down slowly can be converted into high-performance triboelectric layers for TENGs, offering an eco-friendly way to capture energy. The surface microstructures of these bio-wastes are enhanced by cryogenic or chemical treatments, which increases the efficiency of charge transfer and facilitates self-sufficient, sustainable applications.^36,37^ To produce a homogenous solution, human hair waste was chemically treated with ethanolic NaOH and ultrasonically sonicated. This solution was subsequently applied as a thin layer on comparable substrates. On the other hand, waste rubber bands were cleaned and chemically dissolved in methyl ethyl ketone and spin coated or bar coated onto aluminum covered PET substrates to create uniform thin films, which were then thermally dried. A flexible, high performing triboelectric nanogenerator was subsequently created by assembling these rubber bands and human hair films in a face-to-face vertical contact separation setup.^36^ Another separate research revealed after being thoroughly cleaned and dried, human hair debris was cryogenically treated with liquid nitrogen to make it brittle, producing hair particles with higher porosity and surface roughness. The positive triboelectric layer was created by uniformly applying these crushed particles on adhesive copper substrates, and the negative layer was created by coating copper with Kapton. Once fitted together, the device operated in a contact-separation mode, demonstrating a noticeable increase in triboelectric output because of the increased effective surface area and improved charge transfer.^37^ Another novel research showed high-performance TENGs with enhanced charge density and photo response can be created by incorporating carbon nanostructures derived from human hair biowaste into PVDF. These composites give value to bio-waste while enabling motion detection, sustainable energy harvesting, and multipurpose applications.^38^

Fish gelatin from fish waste was used as the positive triboelectric component in a biodegradable TENG. Leaf-inspired microstructures were etched onto the gelatin's surface to increase the contact area and charge production. The lotus leaf-shaped TENG achieved a peak voltage of about 320 V and a current of about 0.8 µA. The device degraded quickly in soil and water and showed exceptional stability over 10 000 cycles. This technique provides a high-performance, economical, and environmentally friendly option for energy harvesting and self-powered sensing applications.^42^

As shown in Fig. 11(a)–(c) the environmentally sustainable, and versatile FG-TENG comprises an Fish gelatin film combined with a polytetrafluoroethylene/polydimethylsiloxane (PTFE/PDMS) composite film. To fabricate PTFE/PDMS composite films, PDMS was first partly processed, then PTFE powder was embedded on its surface, and finally, the thermal curing process was completed at 80 °C fish gelatin films were produced from discarded fish scales *via* a sequence of processes including alkali treatment, acid demineralization, hot-water hydrolysis, and solution casting, followed by drying at ambient temperature. The fish gelatin and PTFE/PDMS films were subsequently trimmed to identical dimensions and bonded with copper foil electrodes and paper substrates. In Fig. 11(d) and (e) the triboelectric performance of FG-TENG showed better result compared to different pairs.^40^

**Fig. 11:**
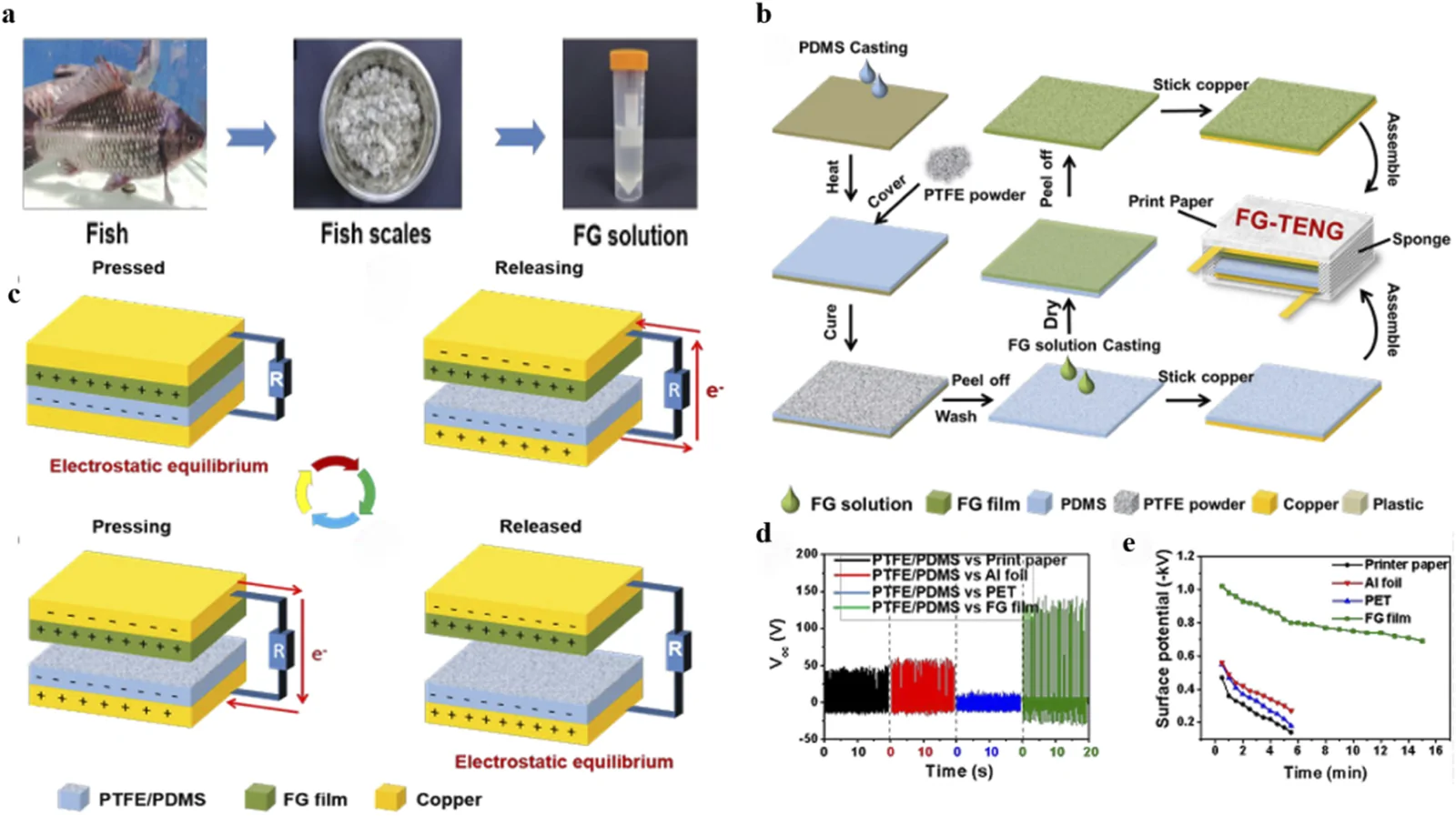
Diagrams depicting the creation techniques of (a) FG solvent, (b) FG-TENG, (c) operational fundamentals of FG-TENG, (d) *V*_oc_ derived from the triboelectric layer of PTFE/PDMS in conjunction with print sheets, foil of aluminum, PET, and fish gelatin films, (e) surface potentials of the above combination. Reproduced from ref. 40 with permission from American Chemical Society,^40^ copyright 2020.

### 3.2. Fabrication of plastic waste-based TENGs

An environmentally friendly method of energy harvesting is the idea of turning used plastic bags into raw materials for the construction of TENGs. In one recent study, triboelectric nano-generator layers were created using plastic bags composed of PA, PVC, and PE plastics. In order to create a conductive electrode, the plastic bags had to be divided into films, fixed to a glass plate, and then covered with a gold coating. Three separate gold-plated PA, PVC, and PE electric plates may then be connected once these films are adhered to an acrylic plate. Pairing the plates and using silver paste to secure a copper wire to the gold electrode created the final spring-based TENG structure.^51,52^ In another work, PTFE and WPB film were used to create a tungsten-electricity TENG device for a pencil-on-paper strain sensor. The WPB-TENG device was assembled using PTFE film (50 µm), WPB film (80 µm), and conductive copper foil.^52^ Furthermore, a straightforward method involved combining a nylon film with a PVDF film without polarization, with the electrodes fabricated through physical vapor deposition (PVD). New electrode materials, including conductive polymers, are under exploration for wired devices. The study also highlighted electrodeless designs such as piezo catalysis and triboelectric designs, showcasing their potential for simplified applications like recycled plastics. By recycling electronic waste (dry cells) to create graphite and plastic bottles, the GP-TENG based on graphite and plastic showed an inventive method. GP-TENG was made using a simple, low-cost, and eco-friendly in-house rapid production method that did not require a clean room, solvents, specialist knowledge, or equipment.^61^

Another interesting project used carbon-coated paper wipes and plastic waste collected from a trash can to create a TENG. An essential part of creating a TENG device with electrodes, PBAT film, copper film, and sandpaper for the triboelectric active layers was a PET waste plastic bottle. An eye-shaped TENG device (EYE-TENG) was created by adding a Kapton layer at the bottom to gather charge.^60^ Using commercial PVC powder, THF solvent, and DBA plasticizer, a PVC gel-based single-layer triboelectric nanogenerator (S-TENG) was created. Stirring the solution and letting it evaporate at room temperature produced a soft, translucent dielectric elastomer during the production process. The S-TENG and tactile sensor were built using the resultant PVC-gel and connected to tactile sensors and electrical cords.^65^ The steps involved in making TENGs from plastic waste are depicted in Fig. 12(a), including PA-PVC-TENG, PA-PE-TENG, and PVC-PE-TENG. To create conductive electrodes, discarded plastic films are cut, positioned on acrylic bases, and lightly covered in gold. The triboelectric nanogenerator was created by combining these films with a spring support mechanism that permits repeated contact and separation. The layered structure of the TENG, which is made up of gold electrodes, plastic triboelectric layers, and the supporting substrate, is shown in a cross-sectional diagram.^51^

**Fig. 12:**
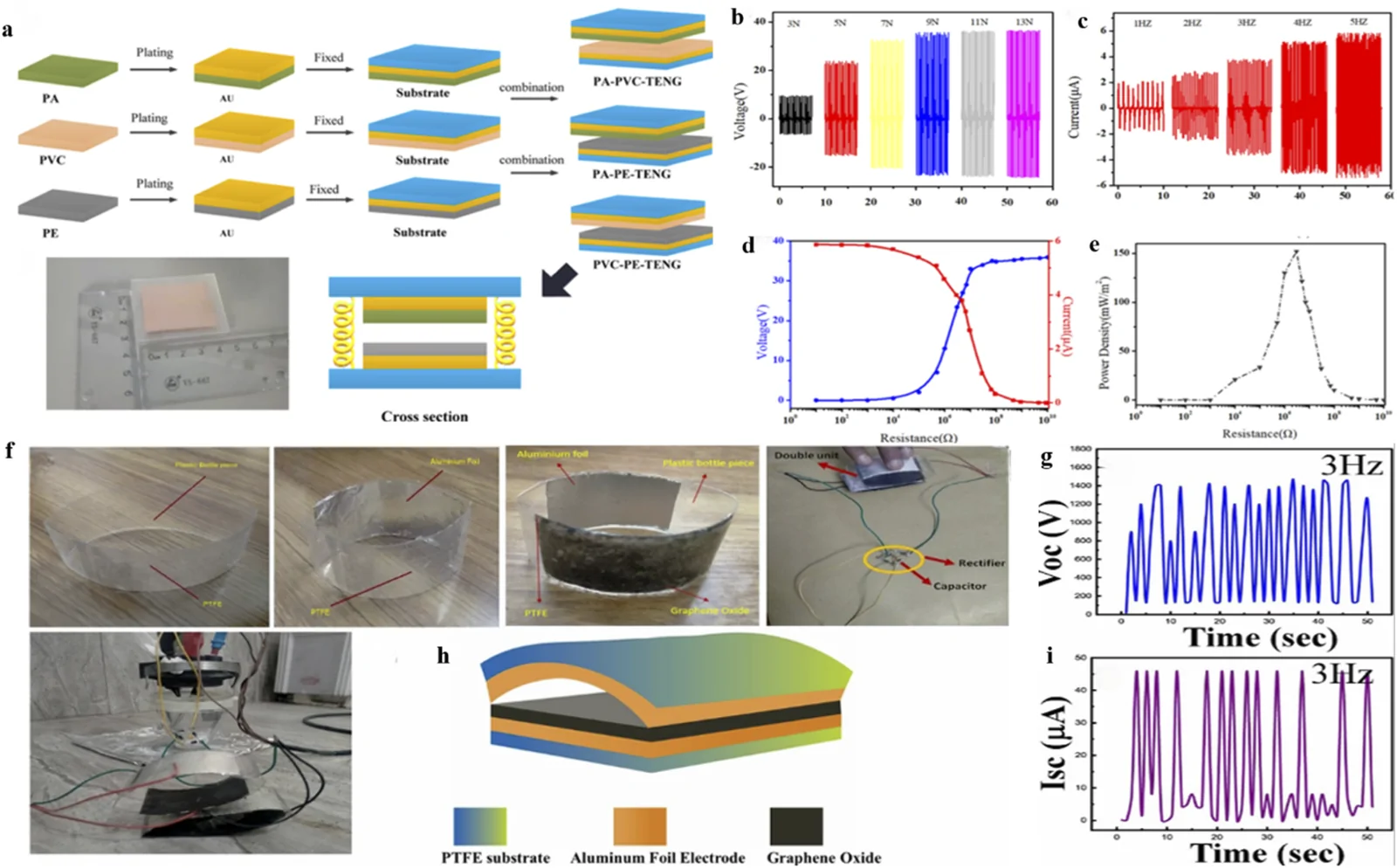
Fabrication of plastic waste based TENG. (a) Construction and design of TENGs made from plastic waste, (b) voltage response at various contact pressures, (c) current variation along vibration frequency, (d) current and voltage in relation to load resistance, (e) output power density compared to resistance. Reproduced from ref. 51 with permission from American Chemical Society,^51^ copyright 2021. (f) and (h) development and structure of SP-TENG, (g) and (i) *V*_oc_ and *I*_sc_ at 3 Hz. Reproduced from ref. 62 with permission from Hexa Publishers,^62^ copyright 2022.

Fig. 12(b) represents the *V*_oc_ of the PA-PVC-TENG increases with augmented contact pressure, indicating that higher pressure improves the effective contact area between the triboelectric films, hence enhancing charge transfer and output performance. On the other hand, Fig. 12(c) appears the *I*_sc_ of the PA-PVC-TENG that exhibits a positive correlation with vibration frequency, indicating that an increased frequency of contact separation cycles enhances the charge flow rate and current amplitude. Fig. 12(d) demonstrates that voltage escalates with resistance while current diminishes and this relationship exemplifies the load dependent behavior of the TENG and facilitates the identification of optimal matching for maximum power extraction. Fig. 12(e) indicates the optimal power transfer point, where current and voltage contributions are equilibrated for maximal energy harvesting.^51^

The full fabrication and structural design procedure for the single electrode SP-TENG device is depicted in Fig. 12(f) and (h). The base of this device is made of readily available PTFE plastic trash bottles, which are covered with a thin 500 µm coating of graphene oxide as the negative layer and aluminum foil as the positive triboelectric layer. After being cleaned, the PTFE sheets are cut into 3 × 6 cm^2^ pieces and, for stability, affixed to the aluminum foil using double-sided adhesive tape. Wires from an abandoned water bath are used to make electrical connections. When the surfaces are tapped with a finger or hand, the gadget produces AC in its contact-separation mode. Practical energy harvesting applications are made possible by a rectifier circuit that transforms the AC output into DC using diodes and a capacitor. At a tapping frequency of 3 Hz, the *V*_oc_ peaks at around 1400 V as shown in Fig. 12(g). More frequent contact separation cycles per unit of time cause the voltage to increase dramatically with frequency, which accelerates the buildup of triboelectric charges on the surfaces of graphene oxide and aluminum. The short circuit current peaks at 45 µA at the same frequency as display in Fig. 12(i). A greater current output is produced during contact separation because the higher frequency speeds up electron transport between the layers. As the tapping frequency increases, the data shows that both voltage and current rise, proving that the SP-TENG efficiently transforms mechanical energy into electrical energy and illustrating how contact frequency affects the device's output performance.^62^

There was a 50 µm-thick PVC film attached to the surface of a sandwich construction using waste polymers like PU, expanded PS, PP, PE foam, and melamine formaldehyde foam (MF), eco-friendly, straightforward, and inexpensive TENGs have been created recently without the need for physical or chemical functionalization. Each TENG was subjected to finite element analysis, which confirmed the experimental findings. An excellent technique for recovering wasted resources for energy harvesting was demonstrated by another study, which used PS extracted from packaging waste to make a TENG.^47,58^ In another work, trifluoroacetic acid, dichloromethane, and PET material were mixed to form the PET solution, which was then used to create a 3D PET aerogel from transparent PET bottles. PPP aerogel and PVDF porous membranes were used in the resulting TENG device, and adsorption tests were performed on the aerogel. The identical setup was used to evaluate the energy harvesting performance.^59^ One noteworthy invention includes the creation of a triboelectric nanogenerator out of inexpensive, easily accessible materials like graphene oxide, aluminum foil, and PTFE waste bottles. Double-sided adhesive tape was used to modify and shape plastic PTFE waste bottles that were gathered from domestic trash for the aluminum foil electrode. Graphene oxide was applied to the TENG device's lower side, serving as a negative TENG layer with electrical connection holes.^62^ Polymer chemistry plays a significant role in determining triboelectric polarity since plastic wastes have generally well-defined polymer structures and specific electron-accepting or electron-donating inclinations.^59,62^

### 3.3. Fabrication of plant waste-based TENGs

The production of TENGs from plant waste signifies a notable progress in sustainable energy harvesting, tackling the escalating issues related to electronic waste and energy sustainability. This innovative approach employs peanut shell powder (PSP) sourced from biowaste peanut outer shells as an economical, plentiful, and renewable triboelectric material. This technique fosters a circular economy and sustainable development by converting agricultural byproducts into functional energy devices and self-sustaining sensors.^69,72^ The eco-friendly biowaste-based PSP-TENG's manufacturing process includes PSP preparation, electrode assembly, and device integration. Triboelectric performance is made efficient and long-lasting by PET-based charge collection, flexible structural support, and uniform PSP film formation. The PSP-TENG's output performance under repeated pressing at 10 Hz where PET act as the electronegative layer and the greatest *V*_oc_ (390 V) and *I*_sc_ (14 µA) were achieved. With asymmetric peaks resulting from pressing and releasing cycles, the improved performance when compared to the device without PET validates the electronegative layer's critical role.^69^

The first stage in producing a PSP-based TENG (PSP-TENG) as shown in Fig. 13(a), (c) and (d) entails mechanically milling dry peanut shells with a high-speed planetary ball mill to achieve a homogeneous particle powder, generally less than 100 µm. A crucial stage entails the uniform application of the prepared PSP onto the electrode substrates, typically accomplished through drop-casting, succeeded by a solvent-free dry-film formation process to establish a consistent active layer. To guarantee surface uniformity and reproducible interfacial contact, any surplus loosely bound PSP particles are meticulously eliminated, often utilizing controlled nitrogen gas flow.^72^

**Fig. 13:**
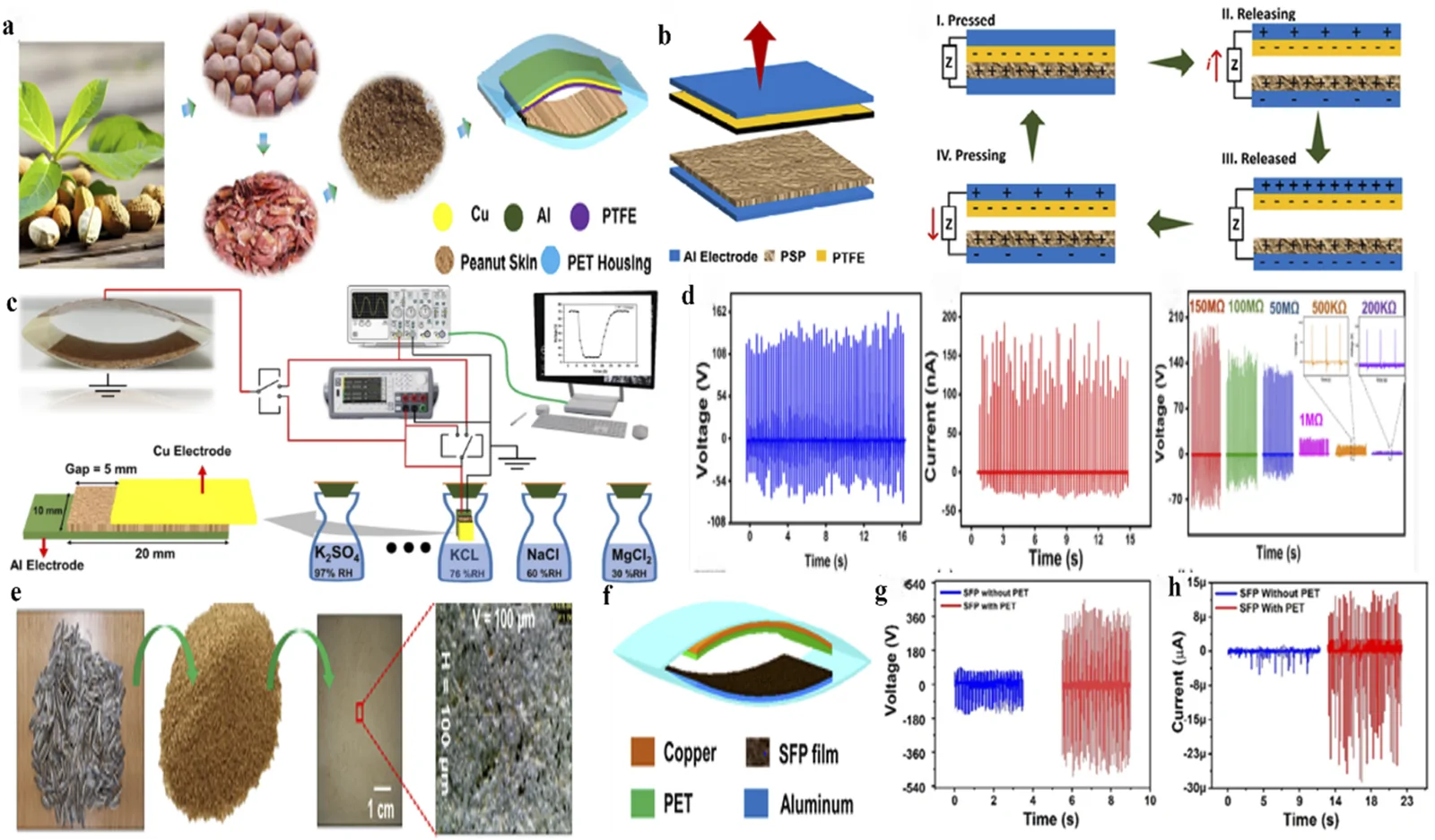
Fabrication of several plant waste-based TENG, (a) PSP-TENG derived from peanut skin powder, (b) working mechanism of contact separation mode PSP-TENG, (c) PSP-TENG device construction and functional description, (d) voltage, current and spectra of voltage at various loading resistances. Reproduced from ref. 72 with permission from MDPI,^72^ copyright 2024. (e) Fabrication of sunflower husks powder based TENG (SFP-TENG), (f) SFP-TENG device, (g) output voltage of SFP-TENG, (h) open circuit current of SFP-TENG. Reproduced from ref. 70 with permission from Elsevier,^70^ copyright 2021.

Fig. 13(b) depicts the four-stage operational mechanism of the PSP-TENG, highlighting charge creation, separation, and electron flow during cyclic compression and release.^72^ The SFP film is made step-by-step in Fig. 13(e), which also shows how to prepare sunflower husk powder, form a thin film, and ensure that the coating is uniform on the Al electrode using optical microscopy. The SFP-TENG device is assembled utilizing PET as the electronegative layer to finish the TENG structure, as seen in Fig. 13(f).^70^ When PET is utilized as the tribonegative layer, the electrical output of SFP-TENG devices with and without PET is compared in Fig. 13(g) and (h), demonstrating noticeably improved performance (488 V, 28.5 µA at 10 Hz). The enhanced output shows outstanding device repeatability and validates PET's increased triboelectric negativity when compared to copper.^70^ The CG-TENG adds a C-coffee ground conducting electrode and a silicone rubber dielectric layer. Layer B, the C-coffee waste electrode, was made by casting a mixture of silicone and coffee waste onto layer A, which is manufactured by putting a silicone mixture to an acrylic plate. A stretchy C-coffee ground electrode is created inside two silicone layers by adding a final silicone layer.^73^

Other studies indicate substantial advancements in the production of high-performance TENGs utilizing plant-based biowaste, in accordance with circular economy concepts and sustainability requirements. Natural rubber (NR), derived from renewable latex, functions as a primary elastomeric matrix, augmented by the strategic incorporation of bio-based nanofillers such as cellulose nanofibers (CNFs) obtained from invasive species. Concurrently, tannic acid, a polyphenolic compound from plant sources serves as a reactive bio-filler in epoxidized natural rubber (ENR), significantly enhancing triboelectric output through improved electron donating capacity. Collectively, these advancements convert low value agricultural residues and biopolymers into durable, eco-friendly TENG platforms capable of powering distributed sensors and wearable electronics, thereby establishing a viable pathway for waste-to-energy conversion in next-generation sustainable electronics.^43,86,87^ To capture waste mechanical energy, a sustainable TENG was developed using a bio-organic material produced from aloe vera (AV). This AV-based device showed exceptional triboelectric and piezoelectric capabilities, reaching a maximum power of 0.42 µW and a peak output of 1.2 V and 200 nA. The TENG's promise for eco-friendly energy harvesting and biosensing applications was demonstrated by its ability to power wearable electronics and function as a self-powered motion sensor.^80^

Another study shows using biocompatible silicone rubber as the tribo-negative layer and aloe vera gel (AVG)@NaCl as an environmentally friendly liquid electrode, a novel liquid single-electrode TENG (LS-TENG) that is both sustainable and morphable was developed. With a output voltage of 157.8 V, JSC of 44.2 mA m^−2^, and power density of 4.61 W m^−2^, the addition of NaCl significantly increased charge mobility and conductivity, producing an unparalleled output performance. With its exceptional sensitivity to even the smallest human movements and its ability to effortlessly adapt to intricate 2D and 3D geometries, this flexible, skin-like technology demonstrated its great promise for use in wearable electronics and health monitoring applications.^122^ The creation of a TENG from rice husks was described in a recent work where SiO_2_ fragments are washed, dried, leached with HCl, and then calcined. Copper and PTFE films serve as electrodes, with PDMS spin-coated on Cu-coated PET forming the positive electrode. The final step includes strategically placing nanoporous RHSiO_2_ particles on the PDMS film.^74^ Another groundbreaking study showed that rice paper and PVC are triboelectrically paired to create a biodegradable TENG. A layer of transparent conductive ink is applied on PET substrates that have been coated with PVC tape to create the RP-TENG, which has remarkable transparency and flexibility. In harsh environmental conditions, this inventive device exhibits mechanical energy harvesting potential.^79^

Recycled tea leaves and discarded aluminum plastic bags were utilized to manufacture a sustainable TENG. Tea flakes or powder were uniformly affixed to aluminum plastic substrates through adhesive bonding and hot pressing. The device was constructed in a contact-separation mode and encapsulated to ensure stable energy harvesting and sensing efficacy. The device delivered a high output performance of 792 V, 42.8 µA, and 488.88 µW cm^−2^, enabling direct powering of LEDs and various office electronics. The developed TAP-TENG demonstrates a simple, low-cost, and eco-friendly strategy for waste recycling, mechanical energy harvesting, and self-powered sensing applications.^80^ The amount of cellulose, hemicellulose, and lignin in plant-derived waste, together with the density of surface hydroxyl and other oxygen-containing groups, all have a direct impact on its electrical performance. Consequently, there might be significant variances in triboelectric output between various plant wastes because of variations in botanical source, chemical composition, surface roughness, moisture sensitivity, and pretreatment.^74,79,80^

### 3.4. Fabrication of medical waste-based TENGs

A recent study that presents an all-flexible waste material-based triboelectric nanogenerator (WM-TENG) is another admirable contribution to the circular economy. The WM-TENG uses recovered Mylar sheets from solid waste and PP from discarded face masks as triboelectric contact layers and a flexible supporting framework that operates in contact separation mode. After thorough testing and study of various disinfection techniques on PP materials, UV-C radiation was chosen for mask cleaning, guaranteeing the retention of electrical energy production.^93^ A study further illustrates the use of medical waste in the production of TENGs by using silicone and discarded X-ray films as frictional layers. After being removed from hospitals, the materials are cleaned, dried, and examined under a microscope before being included into the X-ray sheet based TENG (X-TENG) apparatus. With an active device size of 5 × 5 cm^2^, the X-TENG performs well when attached with carbon tape to aluminum foil electrodes.^95^ The potential of COVID-19 clinical waste, such as masks and gloves, in developing affordable TENG for energy harvesting and sensing applications was highlighted in another work. Electrode-coated mask and glove materials are used in the MG-CS TENG, a polylactic acid (PLA) triboelectric device that is 3D printed. The device's efficiency in energy harvesting and sensing applications is influenced by its dimensions, design, and triboelectric materials.^98^ The detailed process for producing flexible strain sensors and direct current TENG (DC-TENG) from recycled PP substrates derived from used medical masks is shown in Fig. 14(a) and (b). These masks yield a PP layer that is uniformly covered with a conductive ink based on PEDOT : PSS using a simple drop-casting technique. By creating a continuous conductive network, the PP substrate's porous microstructure enhances ink adhesion. The manufacturing of multipurpose wearable electronics is made easier by this ecologically benign and scalable approach. The diagram shows how flexible sensors and self-powered DC-TENG devices are incorporated into one design.^94^

**Fig. 14:**
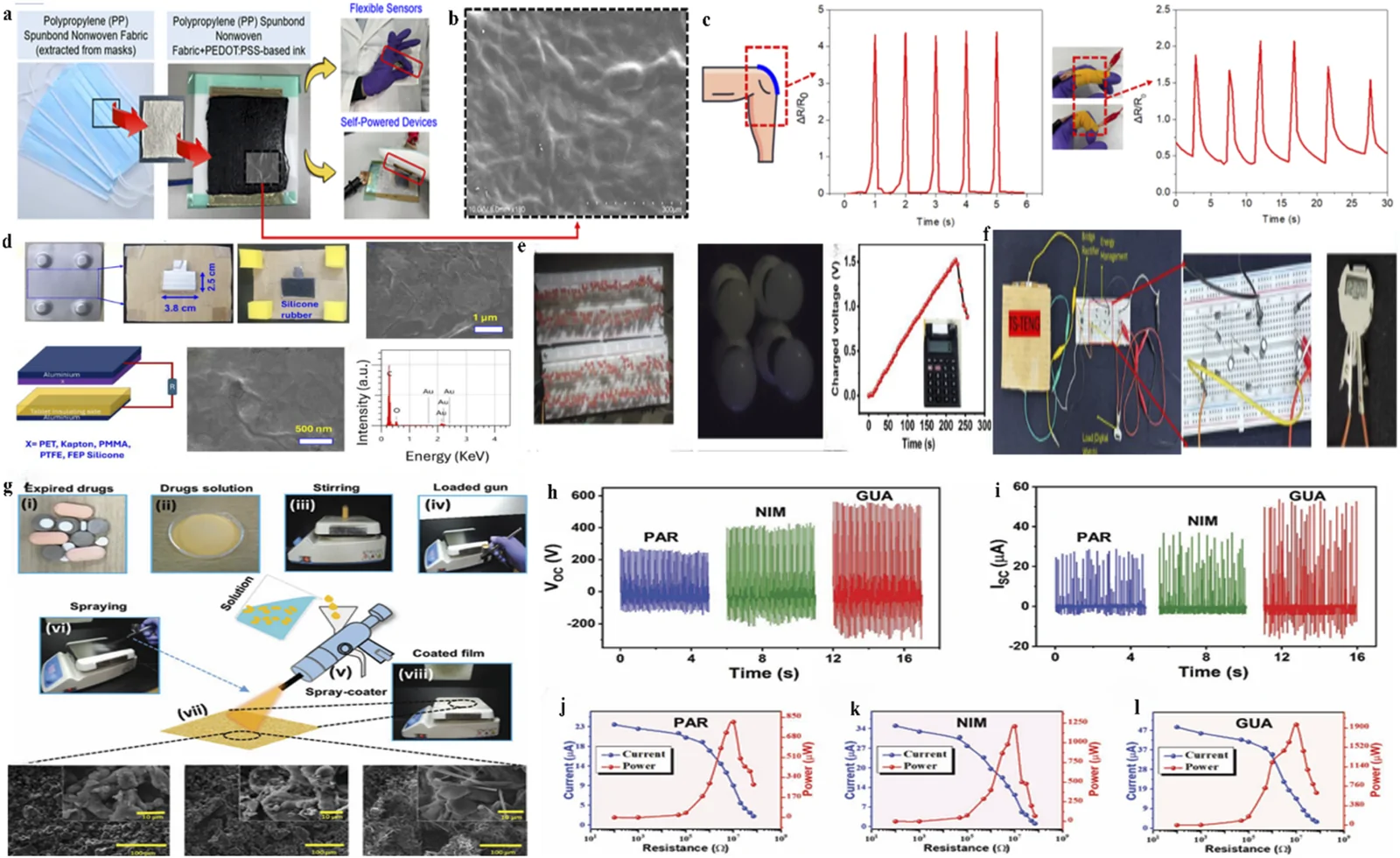
(a) Fabrication and structural characterization of PEDOT : PSS-based wearable devices utilizing recycled medical mask waste, (b) SEM image, (c) effectiveness of PEDOT : PSS/PP flexible strain sensor for wearable motion detection. Reproduced from ref. 94 with permission from Elsevier,^94^ copyright 2025. (d) Tablet blister trash into a possible layer of friction in TENG, (e) image of LED lights and capacitor charging and discharging characteristics in a powering calculator, (f) power management circuit for powering digital watch. Reproduced from ref. 123 with permission from MDPI,^123^ copyright 2025. (g) Production procedure and morphological evaluation of tribopositive films based on expired medications, (h) and (i) electrical output efficiency of expired paracetamol (PAR), nimesulide (NIM), and guaiphenesin (GUA) based TENG, (j)–(l) effect of external load resistance (100 Ω to 100 MΩ) on output current and instantaneous power of expired PAR, NIM, and GUA. Reproduced from ref. 100 with permission from Wiley,^100^ copyright 2021.

The ability of the PEDOT : PSS based PP flexible strain sensor to detect human motion in real time is demonstrated in Fig. 14(c). By displaying clear and consistent variations in resistance, the sensor effectively detects important joint motions, including finger joint motion and knee bending. Without sacrificing performance, its exceptional flexibility guarantees a solid attachment to curved body surfaces. High sensitivity and mechanical durability are demonstrated by electrical signal responses that are clear and constant. These results highlight the potential sensors in human–machine interaction and wearable health monitoring.^94^

In the tablet blister waste-derived TENG depicted in Fig. 14(d)–(f), the tablet blister plastic functions as the principal friction layer, whilst the affixed aluminum layer operates as an electrode. The apparatus effectively produces adequate electrical energy to power 240 LEDs and four LED fixtures *via* continuous hand tapping. The produced energy may be stored in a capacitor and then utilized to power portable electronic devices like calculators and hygrometers.^123^

The complete procedure for producing a TENG using medicinal medication that has expired as shown in Fig. 14(g). This includes dissolving the drug, depositing it *via* spray coating, and forming a thin layer on aluminum substrates. Robust interfacial adhesion and even deposition are guaranteed by the scalable spray-coating technique. FE-SEM pictures of films based on PAR, NIM, and GUA are shown in Fig. 14(g), respectively. Surface roughness and effective contact area are increased by the distinct surface features of these films, which include spherical, rod-like, and aggregated microstructures. The improved microstructural properties increase electrical output by facilitating effective charge transfer during triboelectrification. The *V*_oc_ and *I*_sc_ outputs for TENGs based on PAR, NIM, and GUA are shown in Fig. 14(h) and (i) respectively. The GUA based device exhibits better electrical performance. The output current and instantaneous power density as a function of external load resistance are shown in Fig. 14(j)–(l). The output power first rises and then falls as resistance rises, demonstrating ideal impedance matching, while the current falls. At a load resistance of 10 MΩ, the GUA based TENG reaches a maximum instantaneous power of roughly 1960 µW. These results highlight the superior energy conversion potential and usefulness of triboelectric nanogenerators made from leftover pharmaceutical waste.^100^

Another study reported the selection of medical waste materials and the sequential process for fabricating a TENG solely from recycled medical waste, including aluminum pill boxes, rubber gloves, X-ray films, and paper pill boxes. The constructed human motion sensing TENG (SS-TENG) exhibits notable flexibility and structural integrity, enabling it to adapt to curved surfaces. The physical images demonstrate the compact design of the SS-TENG and the elastic structure of the energy harvesting TENG (SG-TENG).^124^ The literate also discussed the variation in output voltage and current of the SG-TENG with changing external load resistances. As the load resistance increases, the output voltage progressively rises, while the current declines due to a reduction in charge flow. The corresponding output power density, which initially ascends and subsequently descends as resistance increases. The peak power density, approximately 65 mW m^−2^, was attained at an optimal load resistance of 100 MΩ. These results underscore the effective impedance matching and exceptional energy conversion capabilities of the TENG constructed entirely from medical waste.^124^

### 3.5. Fabrication of domestic waste-based TENGs

The straightforward, expeditious, and equipment-free fabrication method facilitates the transformation of domestic waste into functional energy-harvesting devices, rendering sustainable technology accessible to the general populace. This uncomplicated approach markedly diminishes production costs, technical obstacles, and environmental impact, thereby fostering widespread adoption. Such user-friendly fabrication processes pave the way for decentralized, eco-friendly power generation and intelligent self-powered systems in daily life.^63,107,108^

Fig. 15 illustrates the structural design, operational configurations, and electrical performance of TENGs constructed from waste milk carton materials. As depicted in Fig. 15(a), the milk carton features a multilayer composite structure comprising polymeric and paper-based layers, which facilitates efficient triboelectric charge generation through periodic mechanical contact and separation. This layered architecture improves surface charge retention and mechanical durability, rendering the waste material appropriate for energy harvesting applications. The surface charge density and associated current density generated by the milk carton-based TENG are displayed in Fig. 15(b) and (c), respectively. Fig. 15(d) shows the self-charge excitation TENG arrangement, which incorporates an internal charge amplification mechanism to improve output performance. A three-stage voltage multiplier is used in the appropriate simplified electrical circuit, depicted in Fig. 15(e), to gather and magnify the created charges. The self-charge excitation TENG achieves an ideal power output under a matched load state by showing a significant rise in output voltage and power density with increasing external load resistance, as shown in Fig. 15(f). Fig. 15(g)–(i) shows the external charge excitation TENG arrangement. And this externally exciting TENG exhibits higher current density and power production under the same load conditions when compared to the self-charge excitation mode, demonstrating the usefulness of external charge injection in raising energy conversion efficiency.^107^

**Fig. 15:**
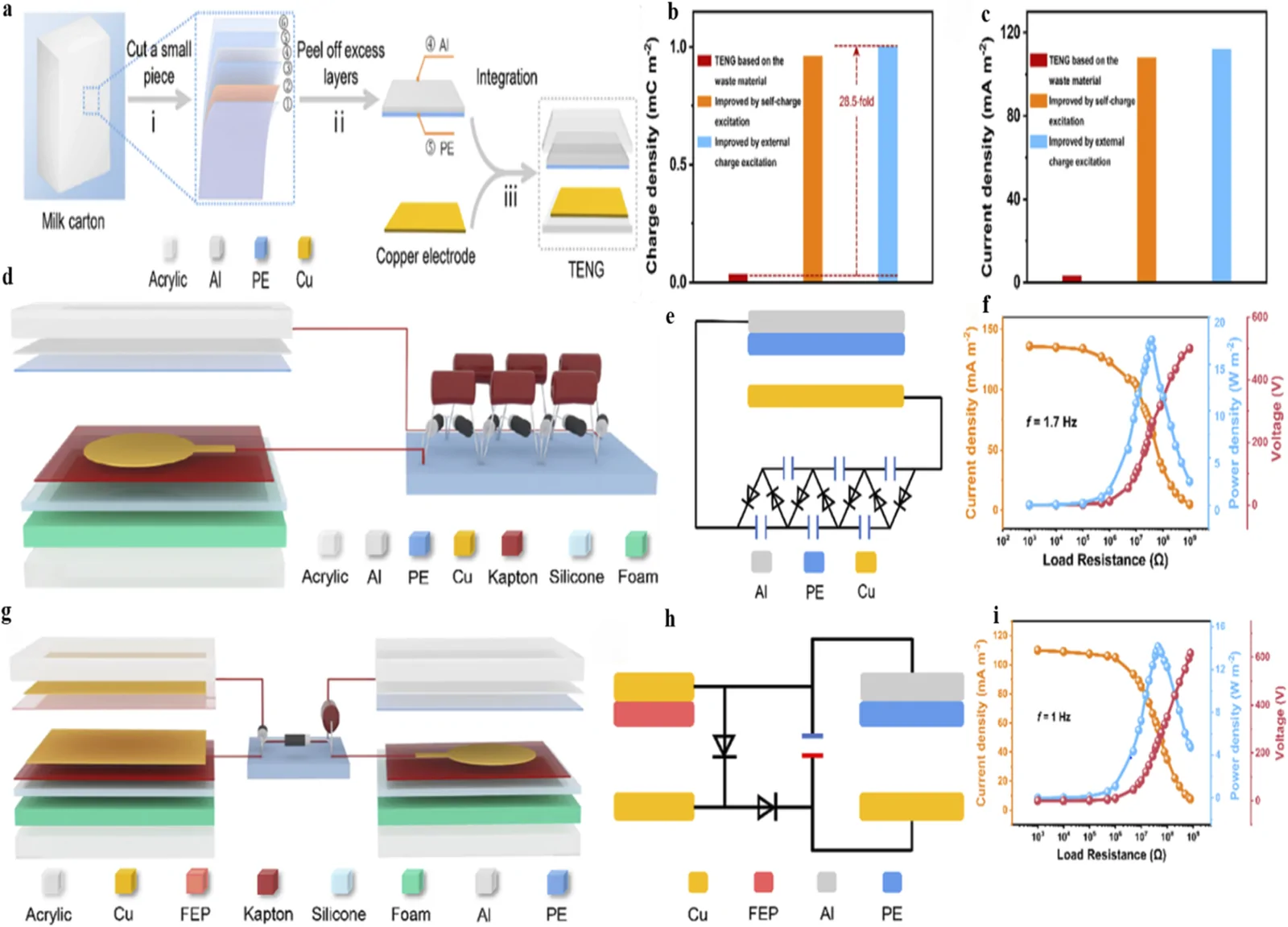
(a) Diagrammatic representation of the waste milk carton's multilayer employed as a triboelectric material, (b) and (c) the surface charge density and current density, (d) the self-charge excitation TENG, (e) circuit diagram of self-charge excitation TENG, (f) current density, *V*_oc_, and power density of self-charge excitation TENG according to various exterior load resistances, (g) external charge excitation TENG, (h) circuit diagram of external charge excitation TENG and (i) current density, *V*_oc_, and power density of external charge excitation TENG according to various exterior load resistances. Reproduced from ref. 107 with permission from American Chemical Society,^107^ copyright 2021.

### 3.6. Fabrication of miscellaneous waste-based TENG

A recent study used LDPEox as the dielectric inductor with ACP to create a TENG as shown in Fig. 16(a) and (b). Copper wires were attached to the aluminum layer in the ACP-TENG, and sheets of LDPE and LDPEox were customized to different diameters. LDPE and LDPEox sheets made up the inner triboelectric contacting surfaces, which formed a sandwich-like structure. The transfer of induced charges was made easier by external copper cables.^111^

**Fig. 16:**
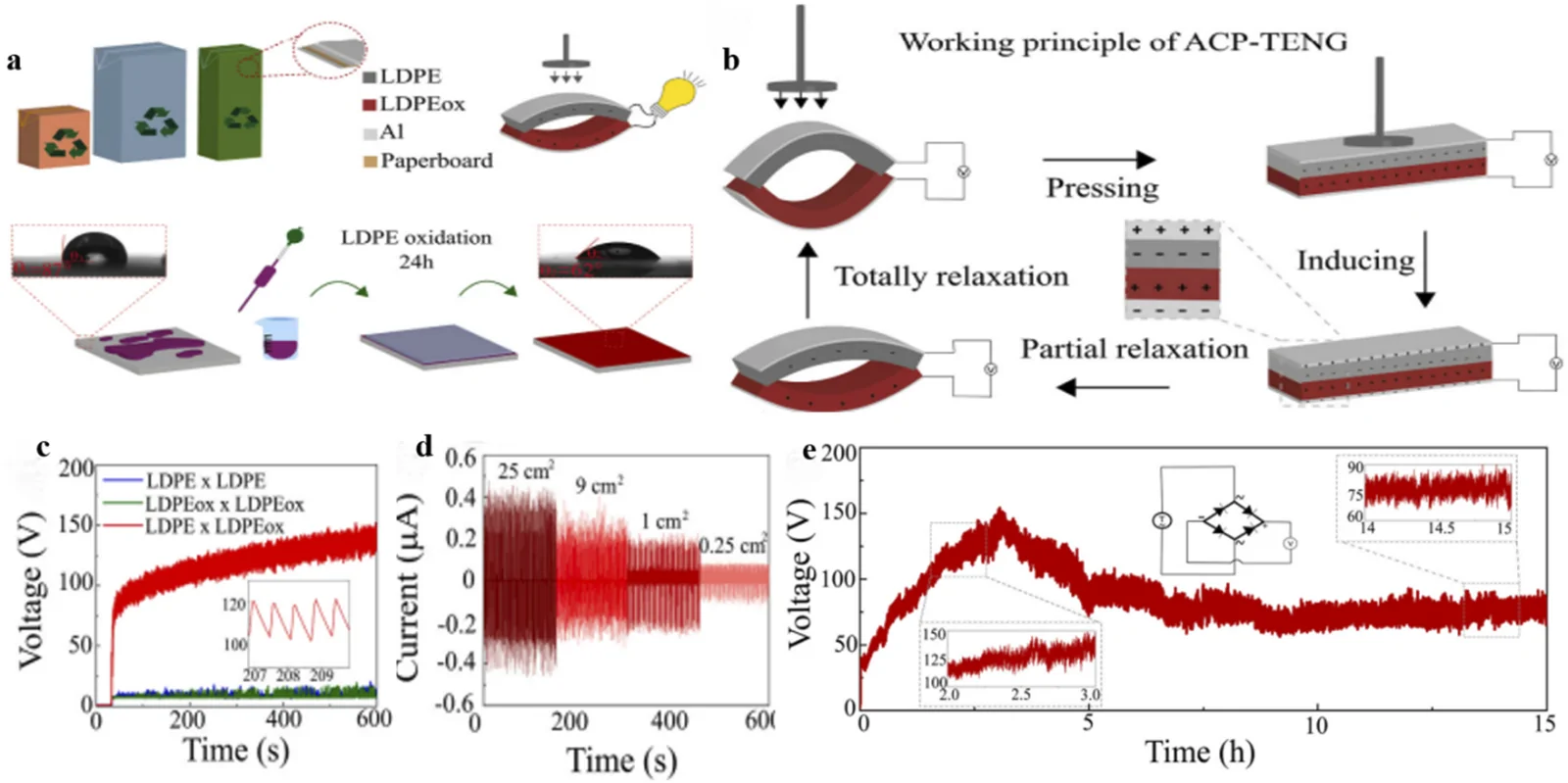
(a) manufacturing process of ACP-TENG employing laminated plastic food packaging, (b) working mechanism of ACP-TENG, (c) and (d) voltage and current of ACP-TENG and (e) pressure-relaxation cycles. Reproduced from ref. 111 with permission from Elsevier,^111^ copyright 2023.

A comparison of the *V*_oc_ generated by ACP-TENG devices built with three different dielectric pairings such as, LDPE–LDPE, LDPEox–LDPEox, and LDPE–LDPEox is shown in Fig. 16(c). Because identical materials have a limited tendency to exchange charges upon contact, designs utilizing LDPE–LDPE and LDPEox–LDPEox produce very low voltage outputs, peaking at about 10 V. This leads to limited electrostatic induction and mild triboelectrification. On the other hand, during cyclic operation, the LDPE–LDPEox arrangement (typical ACP-TENG) produces much greater voltages, up to about 150 V. The difference in surface polarity oxidized LDPE (hydrophilic) gets positively charged, whereas virgin LDPE (hydrophobic) tends to develop a negative charge that is responsible for this enhanced performance. The output voltage is significantly higher due to the enhanced electrostatic induction caused by the increased charge separation. On the other hand, Fig. 16(d) illustrates the *I*_sc_ produced by ACP-TENG devices with varying surface areas. As the device size rises, the amplitude of the *I*_sc_ also escalates. Bigger surfaces facilitate a greater contact area between triboelectric layers, leading to enhanced charge generation and a higher flow of induced electrons through the external circuit.^111^ The pressure-relaxation cycles over time were shown in Fig. 16(e).

The creation of a viscus ink using ethanol, plasticizer triacetin (TA), bPEI, and DMF is the subject of another study. The ink was made by gathering, cleaning, and dissolving used CFs and then cast and oven treated to produce a viscus CA/bPEI/TA film for TENG.^112^

## 4. Material and device engineering strategies

### 4.1. Surface chemistry and triboelectric charge engineering

To improve the electrical performance of TENGs constructed from waste materials, altering the surface is a popular and extremely successful technique. Waste-derived materials frequently feature smooth surfaces, few functional groups, and low intrinsic charge density. Examples of these materials are recycled PET bottles, polypropylene packaging, medicinal saline bottles, leaf powder, textile waste fibers, and carton packaging. These characteristics reduce output performance and restrict effective contact electrification. To increase their triboelectric efficiency, careful surface engineering is therefore essential. Chemical functionalization and physical topographical manipulation are the two main methods that are frequently used.^125–127^ Chemical functionalization entails modifying the surface chemistry of triboelectric materials to adjust their tribopolarity, charge density, and charge retention capabilities.^128,129^ Polymers are commonly employed in TENGs due to their advantageous triboelectric properties and versatility for various modifications. For example, copolymerization of materials such as methoxy poly(ethylene glycol)-poly(ε-caprolactone) (mPEG-PCL) or poly(vinylidene fluoride-trifluoroethylene) (PVDF-TrFE) can alter the dipole density and surface energy, thereby affecting the triboelectric output.^130,131^

Integrating functional groups like amino groups can greatly improve the performance of TENGs by enhancing their electron donating properties. For instance, modifying cellulose by grafting epoxy chloropropane and ethylenediamine to introduce amino groups resulted in a 34.9% increase in output voltage, a 26.7% rise in current, and a 16.7% boost in surface charge density compared to cellulose films without modification. In a similar vein, poly-l-lysine (PLL) has been employed to alter leaf powder and dry leaf surfaces, showing positive effects on TENG output when combined with PVDF, due to the amino groups. Additionally, the molecular engineering of Si (211) surfaces with proton exchangeable organic monolayers has achieved current densities reaching 10^9^ A m^−2^ in sliding Schottky diode TENGs.^49,132,133^

In contrast, physical topography manipulation aims to improve triboelectrification by increasing the effective contact area through surface shape engineering. This is predicated on the idea that contact electrification is a surface phenomenon and that charge generation may be greatly increased by increasing surface roughness and contact sites.^49^ Increasing surface roughness results in more significant charge transfer because it increases the effective contact area during triboelectric interactions. Techniques like etching, sandpaper abrasion, and plasma treatment are employed to produce micro/nanostructures that enhance this effect. For example, it has been shown that adding sandpaper with varying grit levels to silicone rubber strips or aluminum substrates greatly increases TENG efficiency. In particular, compared to an unaltered strip, a silicone rubber strip modified with sandpaper (SRS-TENG) demonstrated a 50% increase in output current, a 40% increase in voltage, and a 20% improvement in transmitted charge.^134^ Surface patterning techniques, such laser etching or engraving patterns that replicate sandpaper or natural surfaces like leaves, produce micro- and nanostructures that improve the effectiveness of friction layers. For instance, chitosan-based composite micro-grating films have greatly improved performance, particularly in medical applications.^49^ In the field of nanoengineering and coatings, advanced techniques utilizing coatings, such as chemically modified nanomaterials or adhesive multilayers, are increasingly employed in TENGs constructed from recycled materials. These methods facilitate the modification of surface energy and chemical interactions, thereby enhancing stability and improving electron transfer rates.^49,135^

Improving the electrical performance of waste-derived TENGs is largely dependent on optimizing surface charge density. The output voltage, *I*_sc_, and power density are strongly correlated with the triboelectric charge amassed at the interface, indicating that augmenting surface charge density can significantly improve device efficiency. In recycled materials, elements like surface imperfections, pollution, and decreased dipole density frequently impede charge formation and retention. Therefore, the key to overcoming these intrinsic difficulties is engineering the surface charge.^49^ According to Paschen's law, the surface charge density of the dielectric friction layer largely determines the output efficiency of TENGs. Limiting air breakdown determines the maximum surface charge that can be achieved and this factor can be successfully increased by raising the dielectric constant.^136^ Multiple investigations have shown that integrating tiny structures or designing functional groups onto the surfaces of triboelectric materials can augment surface charge density.^137,138^ An augmentation in carbon loading results in an increased quantity of carbon nanoparticles on positive triboelectric contact surfaces. This elevation in carbon content effectively expands the surface area in triboelectric contact with the negative (PTFE) triboelectric surface, leading to an enhanced surface charge density. The application of nanomaterials for such performance enhancements is a prevalent practice in TENG designs.^139–141^ Surface roughness and electrical force promote charge exchange, with LDPEox displaying a more complex surface and elevated charge gradients. Even slight alterations in chemical composition can significantly influence the electrostatic charging of PE surfaces, thereby enhancing the efficacy of ACP-TENG.^111^

### 4.2. Conductivity, force and contact area engineering

Augmenting the carbon loading or thickness of the carbon layer significantly improves the conductivity of both electrodes in the C@PW-TENG. Enhanced conductivity promotes efficient charge transfer between the TENG electrodes, yielding a higher electrical output. However, increasing the carbon mass on the PW to 16.23 wt%, despite diminishing the RS of the corresponding C@PW, unexpectedly results in a decline in the electrical output of the respective TENG. This deviation from the expected trend in TENG outputs is ascribed to modifications in the surface characteristics of the triboelectric contact layers.^64^ The conductivity of pure AVG is 0.07 S m^−1^, but when NaCl (0.1 to 3.0 wt%) was added, it rised to 0.25–3.59 S m^−1^ because AVG@1 wt% NaCl based LS-TENG contains improved free ions (Na^+^ and Cl^−^) that means higher output is correlated with higher conductivity.^83,89^

Increased force has the potential to amplify the electrification effect, thereby augmenting energy generation. To assess the real-time energy-harvesting performance of EYE-TENG, a biomechanical energy harvesting analysis was conducted by manually applying pressure to the device through tapping. Under slow external tapping, the device harvested energy at a frequency of 1.95 Hz. Subsequently, an increase in hand-applied force elevated the frequency to 4.377 Hz. Further, fast tapping resulted in a frequency of 4.4 Hz. Additionally, when force was applied through palm tapping, the voltage signal surged to 362 V due to the heightened weight and pressure. Similarly, the current generated from palm tapping reached 26 µA, surpassing the current generated from finger tapping (10.6 µA).^60,69,72^ The improvement in electrical power generation through mechanical force can be attributed to the higher compressibility of the pore structure. As force increases, the contact area expands, leading to a higher electrical output. The current PP-H-TENG and the voltage of SE-TENG exhibited linear increases with the escalating applied force.^43,63,86,87,112,114,122^

In addition to the intrinsic properties of materials, achieving optimal contact at the interface is crucial. The insulating nature of dielectric tribomaterials in TENGs underscores the significance of charge transfer from the triboelectrification layer to the electrode through electrostatic induction. Consequently, effective engineering at the electrode surface, friction layer–electrode interface, and the careful selection of electrodes with suitable work functions and triboelectric materials are essential for increasing surface charge density and power output. Elevated surface roughness plays a pivotal role in augmenting the effective contact area, leading to enhanced triboelectric charge generation.^142,143^ Microstructures and fibers contribute to enlarging the surface contact area, thereby boosting TENG output. The larger contact area induces greater resistive force during droplet transportation. Notably, in TENG devices, the output performance is inversely proportional to the duration of charge transfer, with cool droplets having shorter transportation durations yielding higher output. Therefore, an increased contact area is advantageous for achieving higher TENG output.^144,145^

Expanding the active area of X-TENG (from 3 × 3 cm^2^ to 10 × 10 cm^2^) directly enhances contact electrification, resulting in an increase in output voltage from ∼126 V to 291 V and current from ∼22.7 mA to 81.1 mA.^95^ Augmenting surface roughness in BC composite films improves the contact area between bonded materials. Analysis of surface Young's modulus using AFM indicates that BC composite films with 13.5% particles exhibit a higher modulus than pure BC films. This rise signifies a greater effective contact area due to the ultra-low Young's modulus of PDMS compared to BC composite films.^146^ Consequently, enhanced contact surfaces significantly improve output performance, as corroborated by the established relationship between outputs and contact surface area.^147^

### 4.3. Material modification and electrical parameter optimization engineering

In addition to mechanical force and contact area engineering, notable enhancements in the performance of waste-based TENGs have been realized through material modification and the optimization of electrical operating parameters, which directly affect charge generation, storage capacity, and power delivery efficiency.^33,70,83,86^ The piezoelectric-enhanced effect of CESM contributes to the superior output performance of CESM/ePTFE P-TENG when compared to RESM/ePTFE P-TENG or HESM/ePTFE P-TENG.^33^ Another crucial structural factor is distance separation. Because of the greater potential differences created between dielectric layers during contact-separation motion, extending the separation distance in SFP-TENG devices increases electron transfer efficiency.^70^ Additionally, the drive frequency and load resistance can impact the output performance of TENG. The surface area-to-volume ratio plays a significant role in enhancing output performance, as a larger area increases surface charges during the contact of films, thereby improving total output power. The incorporation of AgNP with NR films enhances the energy conversion of NR-based TENG.

When AgNPs are added to natural rubber (NR) films, their dielectric response is improved. This can lead to increased triboelectric charge accumulation and interfacial polarization, which improves the electrical output of the related TENG.^43,86,87,122^ The generated output can be significantly impacted by structural elements like the active contact area in addition to material-level alteration. The electrical output of the AV film-EH device grew from 4.9 mV and 2.4 nA to 142 mV and 101 nA, and then to 311 mV and 134 nA, by expanding the active area from 4 to 9 and 16 cm^2^.^83^ The greater effective contact area, which offers more interfacial sites for triboelectric charge production and separation, is the main cause of this improvement. Similarly, at a contact frequency of 1.0 Hz, optimizing the NaCl content in the AVG@1 weight percent NaCl-based LS-TENG produced maximum *V*_oc_, *I*_sc_ density, and power density of 114.2 V, 19.1 mA m^−2^, and 2.83 W m^−2^, respectively.^87^ The improvement at the optimal NaCl concentration implies that the triboelectric layer's charge-generation and charge-transfer processes may be influenced by suitable ionic alteration. Therefore, rather of relying just on material selection, these investigations show that TENG performance can be enhanced by a variety of methods, such as dielectric/interfacial polarization, expansion of the effective contact area, and ionic alteration.^83,89^

### 4.4. Electrical configuration and dielectric engineering

The material composition, device area, contact frequency, applied force, and circuit arrangement all have a significant impact on the electrical output performance of TENGs. Voltage, current, and power density are all greatly increased by improving these factors.^43,70,71,86–88^ In TENGs employing NR–TiO_2_, a rise in TiO_2_ filler concentration results in improved electrical output, peaking at 0.5 wt%, yielding 78.4 V and 7.0 µA. However, the enhancement is somewhat constrained due to limited interfacial polarization. Mechanical processes such as ball milling enhance nanoparticle dispersion, increase the effective surface area, and augment dielectric polarization, thereby further elevating electrical output.^70^

In a similar manner, the incorporation of conductive and reinforcing nanofillers, specifically CNF (1.2 wt%) and AC nanoparticles (0.4 wt%), into NR latex markedly improves TENG performance, yielding an approximate fivefold increase in power density. This enhancement is attributed to an elevated dielectric constant, enhanced charge trapping, and superior interfacial polarization.^88^ The addition of AgNPs prepared with CTAB surfactant improves dielectric properties and enhances the electrical output of the NR composite. Conversely, AgNPs without the use of CTAB do not exhibit an improvement in the dielectric properties of the NR composite.^88^

The instantaneous output increased to 600 V and 40 µA when the top and lower AS-TENG units were connected in series. This is roughly double the voltage amplitude of a single unit. On the other hand, the parallel arrangement increased the current to 80 µA while producing 300 V.^106^ These findings show that rather than enhancing the material's inherent capacity to generate triboelectric charges, series and parallel connections mainly offer distinct paths for voltage and current scaling.^108^ In a different method, PVC-gel treatment boosted the S-TENG's electrical output by about 20 times when compared to native PVC. Stronger negative triboelectric properties and a 190-fold rise in dielectric constant were linked to this improvement, which can encourage charge accumulation and boost the ensuing electrical output.^65^ Therefore, the two methods enhance device performance through distinct mechanisms like PVC-gel treatment alters the intrinsic dielectric and triboelectric characteristics of the active material, whereas electrical configuration allows output matching by circuit-level integration.^65,108^

### 4.5. Mechanical robustness and structural flexibility engineering

Enhancing the mechanical characteristics of waste-based TENG is a critical step in improving their functionality. By increasing the tensile strain capability from 0% to 300%, the TENG demonstrates exceptional stretchability, allowing it to adapt to various deformations and movements. The utilization of a highly stretchable (700%) and transparent (87–97%) biocompatible silicone rubber (SR) film as the tribo-negative and packaging material in AVG@NaCl-based LS-TENG contributes to structural integrity even during extreme deformations, such as folding, rolling, twisting, and stretching. The transparency levels ranging from 72% to 91% further enhance the device's versatility.^122,148,149^ Additionally, incorporating a CA/bPEIpolyionic skin film in SE-TENG showcases improved mechanical properties, including an 8 MPa strength and 102% elongation. This represents a 20% strength decrease and a remarkable 143% elongation increase compared to CA alone. The addition of bPEI molecular chains enhances the softness and elasticity of the CA film, contributing to the overall mechanical improvement of the TENG.^112^

### 4.6. Nanoparticles engineering

Nanoparticles have been employed as a strategic enhancement to improve the functionality of waste based TENG. In the case of TiO_2_ nanoparticles, prolonged ball-milling times led to their disintegration, resulting in well-dispersion within the NR polymer matrix. This improved dispersion contributed to enhanced TENG performance. The high dielectric constant of TiO_2_ makes it an effective filler for improving the dielectric constant of NR composite films.^88^ For C@PW-TENGs, the gradual increase in carbon mass loaded onto the PWs (ranging from 0 to 15.13 wt%) led to a corresponding enhancement in short circuit charge, *I*_sc_, and output voltage values. The maximum values reached were approximately 78 nC, 1.56 µA and 80 V, respectively.^150^ In the case of RH_SiO_2__ particles, an enzymatic treatment resulted in terminated bonds (–NH_2_, –Cl, –F) on the surface, modulating electronegativity and leading to a 56.67-fold increase in charge density for RH_SiO_2__ TENG compared to untreated RH_SiO_2__ TENG. Subsequent FOTS treatment further increased power density from 0.077 mW m^−2^ to 261 mW m^−2^, representing a remarkable ∼339 000% enhancement.^151^

### 4.7. Other engineering strategies

Efforts to enhance the functionality of waste-based TENG include deliberate adjustments to the triboelectric film thickness. Generally, reducing the thickness of the triboelectric film leads to an increase in surface charge density. The operation of the TENG involves the transfer of charges, and as the film thickness increases, more charges may be transferred, particularly due to the increased charges of the fish gelatin film. This correlation explains the observed enhancement in output performance with an increase in film thickness.^152^ Furthermore, the integration of the TENG with both self-charge excitation and external charge excitation circuits significantly improve charge densities.^107,108^ However, it's worth noting that in the case of RP-TENG, an increase in the thickness of rice paper leads to a decrease in electrical output.^79^ In the analysis of triboelectric potential variations in RH_SiO_2__ films with different pore numbers, theoretical calculations using COMSOL software were conducted, considering practical rice husk morphology (5 × 5 cm^2^ area).^151^ The triboelectric potential increased from 634 V (bump arrays) to 2336 V (increased porosity in bump-arrays SiO_2_ film). The rise in dielectric constant from 2.7 to 3.9 in RH_SiO_2__ films enhances induced electrostatic charges through the triboelectric effect, resulting in higher potential compared to raw rice husks.^74^

## 5. Application of waste-based TENGs

### 5.1. Powering

The rapid expansion of portable and wearable electronics has created an imperative demand for sustainable, lightweight, and autonomous energy solutions. Conventional batteries are constrained by their finite lifespan, environmental concerns, and the requirement for frequent replacement, which restricts their prolonged application in distributed and wearable systems.^153,154^ This scenario illustrates that TENGs constructed from waste materials offer a viable method for converting ubiquitous mechanical energy into usable electrical power while addressing plastic waste management challenges. Due to their chemical stability, flexibility, and advantageous triboelectric characteristics, plastic bags emerge as attractive materials for fabricating waste-derived TENGs. Their intrinsic capacity to donate or accept electrons during contact-separation processes renders them particularly efficient in charge generation. By repurposing discarded plastic bags as triboelectric layers, it becomes feasible to develop cost-effective and environmentally sustainable energy harvesters capable of powering low-energy devices. TENGs exhibit significant potential for powering portable electronics, including digital calculators, electronic watches, and wearable sensors. The integration of recycled plastic materials into TENG designs enhances sustainability while maintaining sufficient output performance for practical application in everyday electronic devices.^51,52^ Furthermore, sophisticated material combinations such as PET–Cu composite films and NR–TiO_2_ nanocomposites have expanded the functional capabilities of waste-derived TENGs. PET–Cu films augment electrical conductivity and charge transfer efficiency, whereas NR–TiO_2_ enhances dielectric properties and surface charge density. These material innovations facilitate the generation of stable electrical output, appropriate for powering compact devices, including wristwatches and selecting low-power medical apparatus.^31–33,64,70,88^

### 5.2. Automotive, robotics and energy harvesting

The integration of waste-derived TENGs into automotive systems, robotics, and energy harvesting represents a significant advancement towards sustainable, self-sufficient solutions that address environmental waste challenges and energy requirements.^155,156^ TENGs sourced from waste are strategically employed in the automotive sector to harness mechanical energy from vehicle motion. A high-performance TENG constructed from recycled tire rubber, reinforced with styrene–butadiene rubber (SBR), has exhibited exceptional durability and reliable output under repeated mechanical strain, rendering it ideal for application in tire treads or suspension system.^155^ These devices convert rotational motion, vibrations, and road impacts into electrical energy, capable of powering wireless tire pressure sensors, brake wear monitors, or lane-departure warning systems without requiring battery replacements. This approach is consistent with the overarching trend in intelligent transportation systems, where TENGs serve as both power sources and highly sensitive sensors for real-time vehicle condition monitoring.^156^

In robotics, waste-derived TENGs are transforming the design of self-sustaining tactile and proprioceptive systems. Flexible TENGs constructed from recycled polystyrene infused with tea-leaf powder demonstrate increased surface charge density attributable to the natural cellulose and polyphenol constituents of the biofiller, leading to superior voltage output and mechanical durability.^157^ These materials are especially appropriate for soft robotic skins, serving as distributed pressure sensors that produce electrical signals in reaction to contact, deformation, or shear forces. For example, a TENG-integrated robotic gripper can autonomously sense object slippage and modify grip strength in real time, negating the necessity for external power or intricate signal processing units.^158^ Moreover, TENGs generated from medical waste, such as repurposed X-ray films, have exhibited adequate sensitivity to detect small human movements, including finger flexion and walking patterns, facilitating their application in wearable exoskeletons or rehabilitation robots.^95,124^

Many waste polymers, such as PET and LDPE, are biocompatible and flexible, which facilitates their integration into human–robot interaction interfaces where comfort and safety are crucial. In the field of general ambient energy harvesting, waste-based TENGs take advantage of the ubiquitous presence of mechanical movements, such as wind, rain, footsteps, and ocean waves, to generate usable electricity. By increasing contact area and ion adsorption, cellulosic waste materials, such as cotton textiles and paper byproducts, have been converted into porous triboelectric layers with enormous surface areas that significantly improve charge production.^159,160^ For example, a TENG constructed from leftover sportswear materials can provide enough energy during exercise to power GPS units or heart rate monitors.^161^ Similarly, carbonized waste products from plastic pyrolysis or biomass are employed as tribo-positive layers or conductive back electrodes to increase power output density and electron transfer efficiency. A fully recyclable TENG constructed from soda cans and single-use cups is one recent example. It achieves an *V*_oc_ of more than 300 V, sufficient to power tiny LEDs or charge capacitors for sporadic sensor use.^13^ Moreover, creative applications extend beyond the production of electricity. For example, a waste-plastic TENG has been used to power a mechano-electrochemical process that produces defect-free graphene, showing how these devices may support green chemistry without depending on grid electricity.^26^

### 5.3. Biomedical application

The realm of biomedical applications witnesses significant advancements with the integration of TENGs, particularly exemplified by ESM-based TENGs. Electrospun Membrane (ESM)-based TENGs demonstrate substantial promise within the biomedical field, showcasing their capability to power wearable and self-powered electric gadgets. This innovation opens new avenues for the development of biomedical devices that can operate independently without the need for traditional power sources. Adding to the array of biomedical applications, the ostrich EM-TENG, detailed in a noble study, emerges as a noteworthy technology. Functioning as a smart sensor in wearable electronics, this TENG specifically contributes to biomedical monitoring and motion sensing. The unique features of the ostrich EM-TENG make it an asset in various healthcare applications, offering real-time data for monitoring physiological parameters and detecting movement patterns.^31–33^

As shown in Fig. 17(a) the completely biodegradable and water-soluble TENG (WS-TENG), created from recycled wastepaper, may track human physiological signals, such as respiration, in real time with high sensitivity and flexibility. Its lightweight, biocompatible, and skin-friendly properties allow the WS-TENG to be directly affixed to the human body as a disposable bandage sensor.^162^ The biowaste-derived TENG as shown in Fig. 17(b) created from cellulose, chitosan, and gelatin gained from wood waste, crab shell and fish scale respectively exhibits considerable promise for biomedical and oral healthcare applications, functioning as a self-powered bite sensor to monitor oral chewing force and dental problems in real time. The device can identify irregularities, including fractured teeth and dental misalignment, by analyzing variations in electrical output during biting.^163^ A pioneering study as shown in Fig. 17(c) created a 3D network layer type TENG sensor by carbonizing dry loofah sheets, capable of harvesting energy from diverse body movements and monitoring real-time fluctuations in physiological signals, including normal breathing *versus* tachypnea, facial expressions, pulse rate, vocal data, and motions of the fingers, wrists, and elbows.^24^ In Fig. 17(d) BD-TENG was constructed utilizing biodegradable polymers including PLGA, PCL, PHB/V, and PVA, in conjunction with magnesium electrodes, facilitating complete degradation within the body post-function. The BD-TENG harvested biomechanical energy from bodily movements, producing an output voltage of approximately 40 V and a current of around 1 µA, adequate for powering temporary implantable medical devices. In biomedical applications, the produced pulsed electric field effectively directed nerve cell growth and orientation and potential for neural tissue repair and regeneration. The implantable device also displayed remarkable cytocompatibility and did not induce significant infection or adverse tissue response during *in vivo* studies in rats.^164^

**Fig. 17:**
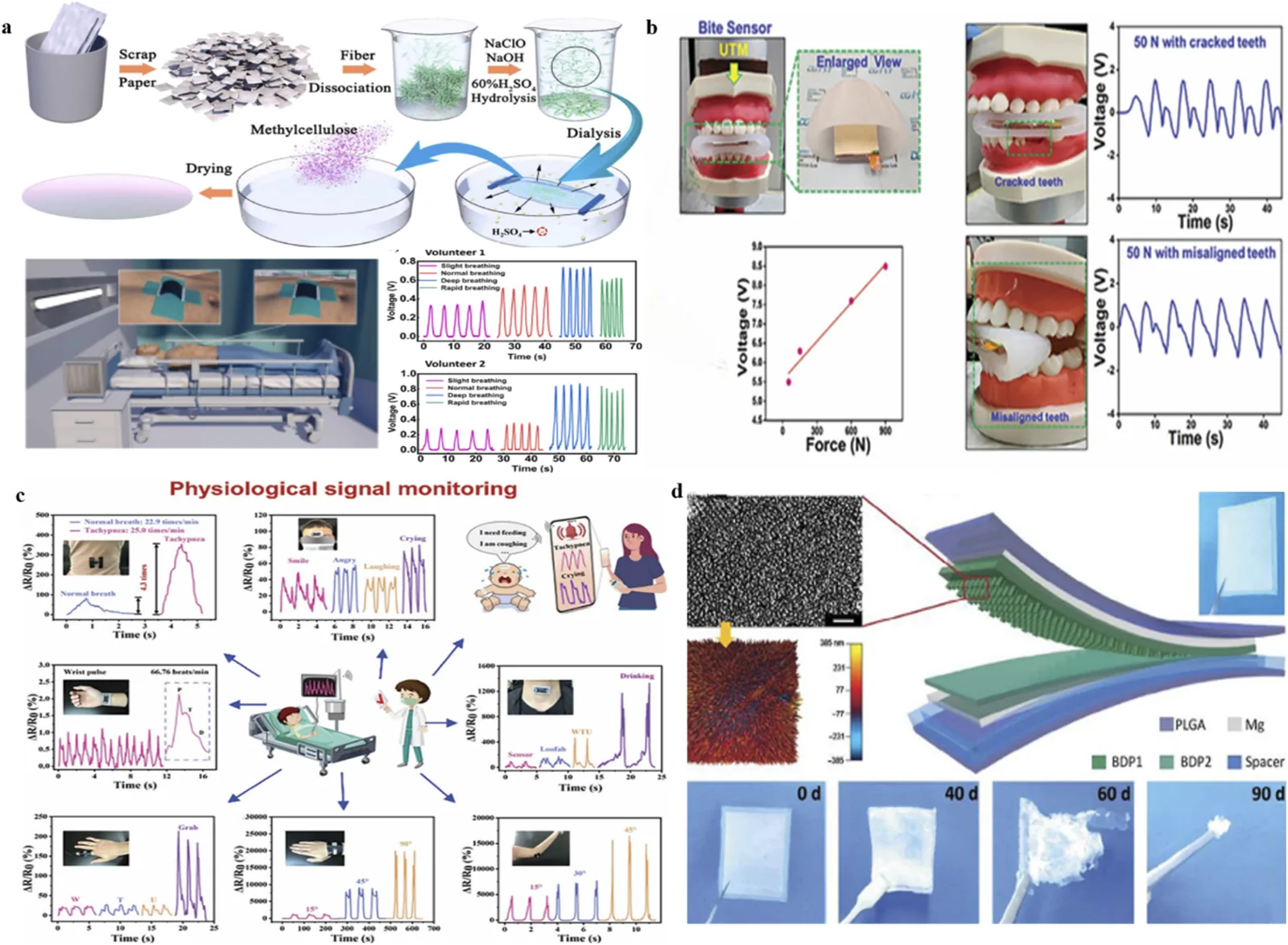
(a) Real time respiration monitoring by biodegradable water soluble TENG. Reproduced from ref. 162 with permission from Elsevier,^162^ copyright 2022. (b) Monitoring dental health using TENG based on food waste, crab shells and fish scale, (c) the carbonized loofah TENG sensor uses self-powered strain sensing to detect human motion. Reproduced from ref. 24 with permission from Wiley,^24^ copyright 2024. (d) Biodegradable TENG as an implantable power source. Reproduced from ref. 164 with permission from American Association for the Advancement of Science,^164^ copyright 2016.

Furthering the impact in biomedical settings, the biodegradable TENG (BD-TENG), highlighted in another study introduces a transformative approach by converting biomechanical energy into electrical power. This TENG serves as a temporary *in vivo* medical power supply, illustrating its potential to harness the body's movements and translate them into a sustainable energy source. The BD-TENG, with its capacity for *in vivo* applications, represents a notable advancement in the field of temporary medical power supplies, offering a biocompatible and efficient solution for diverse biomedical scenarios. Collectively, these biomedical applications underscore the evolving role of TENGs in enhancing healthcare technologies and powering innovative solutions for improved patient care.^14,165^

### 5.4. Eco-friendly wearables sensors

The landscape of eco-friendly wearables witnesses significant strides with the integration of innovative TENGs, exemplified by PSP-TENG. The PVDF and Sponge-like Porous Polydimethylsiloxane (SPPD) based TENG, abbreviated as PSP-TENG, stands out for its unique ability to utilize biowaste as a power source. This groundbreaking technology not only addresses environmental concerns by repurposing biowaste but also promotes sustainability in the wearables sector. By converting biowaste into electricity to power LEDs, the PSP-TENG contributes to the development of eco-friendly wearables, emphasizing the potential for environmentally conscious solutions in consumer electronics.^69^

Additionally, the SFP-TENG outlined in another study adds another dimension to the eco-friendly wearable landscape. Serving as a biomechanical energy collector, the sustainable and flexible piezoelectric TENG detects vibrations and translates them into electrical power. This capability enables the SFP-TENG to power small gadgets such as stopwatches, showcasing their versatility in harnessing energy from human movements. As a result, the SFP-TENG not only offers a sustainable solution for wearables but also highlights the potential for self-powered devices in various applications. Together, PSP-TENG and SFP-TENG exemplify the transformative impact of TENGs in shaping the future of eco-friendly wearables, blending technological innovation with environmental consciousness. TENGs are making significant strides in the realm of touch and pressure sensors, each serving unique purposes within this innovative landscape.^70,71^ The TAP-TENG stands out for its ability to detect various touching states. This includes not only discerning different touches but also harvesting energy from activities like book flipping and drawer pulling. The dual functionality of touch detection and energy harvesting underscores the versatility of TAP-TENG, showcasing its potential in creating self-powered systems that respond to human interaction.^80^ Complementing this, CG-TENG sensors emerge as pioneers in monitoring physiological signals. These sensors, based on contact-separation mode TENGs, offer a self-powered solution for diverse applications requiring real-time physiological data. By harnessing the triboelectric effect, CG-TENG sensors provide an energy efficient means of monitoring human physiological signals, paving the way for innovative developments in healthcare and wearable technologies.^73^ Furthermore, the S-TENG, introduced in recent studies revolutionizes tactile sensing by enabling it without the need for traditional electrode grid layouts. The stretchable TENG, with its unique design and material properties, offers a flexible and responsive solution for detecting touch and pressure. This innovative approach to tactile sensing enhances the possibilities for creating self-powered devices in various sectors, from consumer electronics to medical applications. In essence, these TENG-based touch and pressure sensors showcase the transformative potential of triboelectric technology in advancing interactive and energy efficient systems.^34,35,43,87,88^

### 5.5. Environmental monitoring and alert systems

A paradigm shifting combination of sustainable materials engineering, autonomous sensing, and real time ecological surveillance is represented using waste-based TENGs in environmental monitoring and alarm systems. These devices reduce reliance on traditional power sources and reduce environmental pollution by repurposing globally abundant waste streams, such as post-consumer plastics, medical discards, marine biowaste, and agricultural residues, into functional energy harvesting and sensing platforms.^91,114^ The TENG made from used CFs, a ubiquitous global pollutant made of cellulose acetate, is a fundamental example. This gadget uses plastic trash as the tribo-negative counterpart and the fibrous microstructure and intrinsic surface polarity of CFs as a tribo-positive layer. The resultant CF-TENG attains excellent output performance, producing enough power to operate low-energy sensors and acting as a PM detector in the air. PM causes detectable changes in *V*_oc_ and *I*_sc_ as it builds up on the filter surface, changing the local charge distribution and dielectric characteristics. This allows for real-time air quality evaluation without the need for external power.^114^ Another attractive option is marine biowaste. For the first time, seashells rich in crystalline calcium carbonate with naturally occurring hierarchical microstructures have been used as a triboelectric material, producing a TENG with an astounding power density of 948 mW m^−2^. When used as submerged sensors or floating buoys in riverine or coastal settings, these devices concurrently monitor water quality metrics and capture energy from wave motion. Variations in turbidity, pH, or salinity alter the dielectric constant at the triboelectric interface, changing the output characteristics in a measurable way. This makes it possible to conduct continuous, battery-free surveillance to detect algal blooms, chemical spills, or illicit discharges early on, which is essential for protecting ecosystems and sending out public health advisories.^91^ AS-TENG plays a pivotal role in powering landslip monitoring systems, serving as an early warning mechanism for potential landslides in natural environments. This application highlights the TENG's capacity to contribute to environmental safety by providing a reliable power source for monitoring equipment in remote or challenging terrains.^100,104^

### 5.6. Biodegradable and self-powered systems

Integrating waste derived materials into TENGs presents an innovative approach for sustainable energy harvesting, particularly within biodegradable and self-sustaining systems. This strategy addresses the global challenges of electronic waste accumulation and the demand for renewable energy by converting discarded materials, such as agricultural byproducts, food remnants, used textiles, and bioplastics, into efficient energy-harvesting devices. The core principle relies on the triboelectric effect, wherein electrical charge is generated through the contact and separation of two distinct materials, with a notable advancement and the use of naturally degradable or recycled materials as the active triboelectric layers. This TENG not only offers a green alternative but also holds promise for diverse applications in the medical field, aligning with the growing emphasis on environmentally friendly and biocompatible technologies.^64,107,108,166^

In Fig. 18(a) the fish bladder film based TENG (FBF-TENG) functions as a self-sustaining system, serving as a wireless intelligent switch. The instrument performs as a non-contact position and distance sensor, with the output voltage varying in accordance with the separation distance between the fluorinated ethylene propylene (FEP) film and the FBF-TENG surface.^24,167^ FRF-TENG exhibited in Fig. 18(b) was synthesized utilizing phytic acid and saccharose derived from rice bran and sugarcane inside the KIT-6 framework for applications in flame retardancy and posture detection. The manipulated RSSP-TENG as shown in Fig. 18(c) was successfully applied to power electronic equipment and intelligent sensing technologies which exhibit high electrical performance, scalability, transparent nature, and exceptional biological compatibility, rendering it suitable for wearable and implantable electronics. Furthermore, the RSSP based patch demonstrated robust antibacterial efficacy against both Gram-negative and Gram-positive bacteria through electrically induced sterilization without the use of pharmaceuticals.^168^ The engineered flexible nanogenerator based on orange peel and PVDF as shown in Fig. 18(d) generated an *V*_oc_ of approximately 90 V and a peak power density of around 135 µW cm^−2^ through basic finger pressing. It was also proficient in harvesting mechanical energy from routine human activities, including bending, twisting, walking, and sliding door movements.^169^

**Fig. 18:**
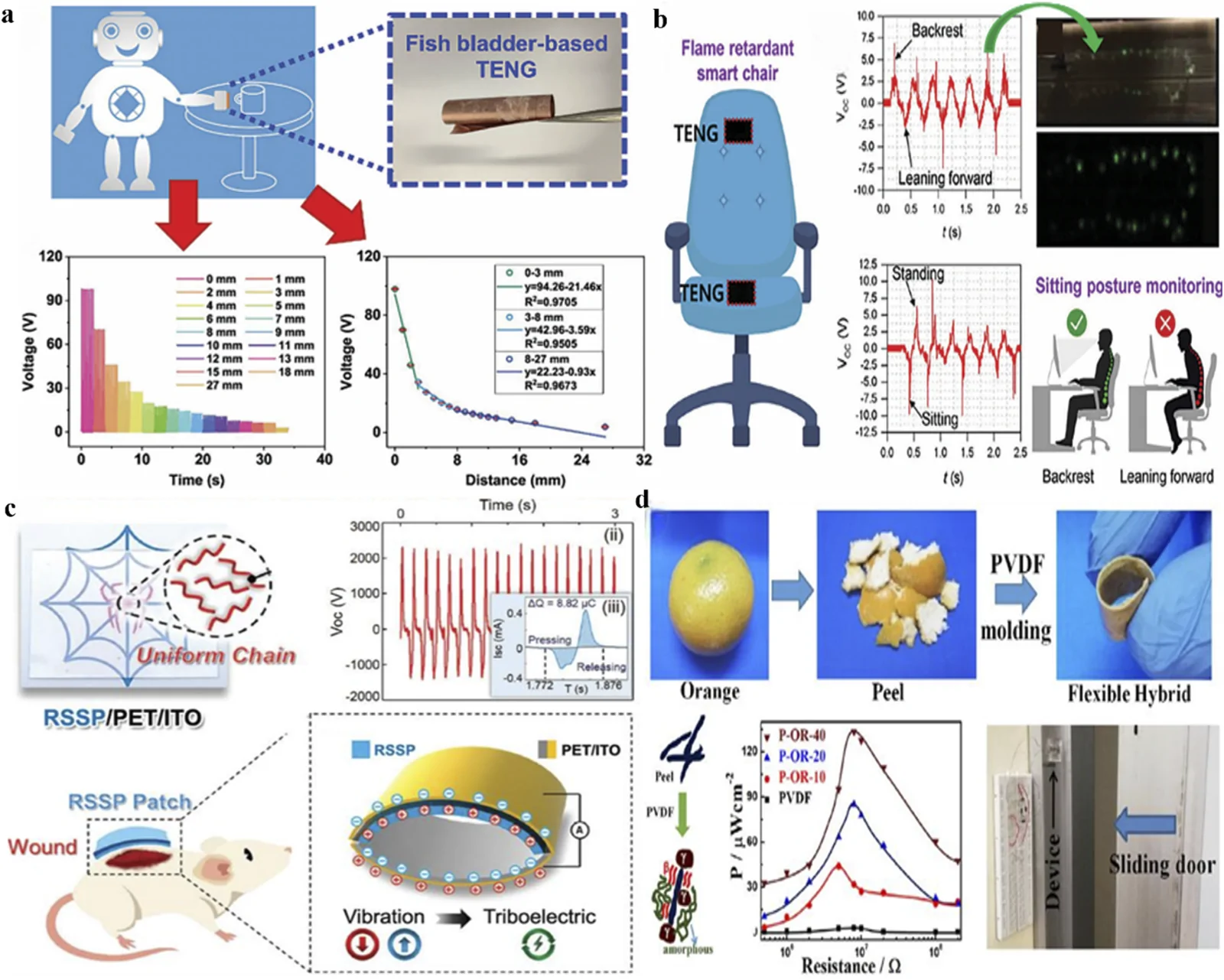
(a) Schematic diagram of FBF-TENG and their potential application as a sensor, (b) TENG based on rice bran and sugarcane waste for monitoring sitting position and flame retardancy. Reproduced from ref. 24 with permission from Wiley,^24^ copyright 2024. (c) TENG from spider silk (RSSP-WL-TENG). Reproduced from ref. 168 with permission from Wiley,^168^ copyright 2018. (d) Energy harvester utilizing orange peel. Reproduced from ref. 169 with permission from Elsevier,^169^ copyright 2020.

In a parallel trajectory, the RP-TENG stands out for its exceptional recyclability and contribution to self-powered electronic systems. By harnessing mechanical energies to power LEDs, the recyclable paper-based TENG showcases a sustainable approach to electronic device power sources. This innovation not only addresses environmental concerns but also presents a tangible solution for advancing self-powered electronic systems with an eco-friendly footprint.^47,56,58,59,61,67,74^ Additionally, the WM-TENG, highlighted in plays a pivotal role in addressing plastic pollution and meeting electricity demands in a sustainable manner. Functioning as an adaptable and cost effective nanogenerator, the wood microstructure based TENG provides a renewable energy solution. By repurposing waste materials and contributing to sustainable power generation, the WM-TENG exemplifies the potential of TENGs in both environmental conservation and meeting the escalating global demand for electricity. Together, these TENGs showcase the intersection of biodegradability, self-powering capabilities, and environmental consciousness, heralding a new era in sustainable energy systems. Diversifying its applications, TENGs exhibit versatility in miscellaneous domains, contributing to advancements in electronic devices and waste repurposing.^93,94,112^

### 5.7. Miscellaneous applications

The X-TENG takes center stage by powering LED displays and electronic gadgets through the utilization of charged capacitors. This innovation underscores the adaptability of TENGs in meeting the power demands of various electronic components, offering a sustainable solution for energy needs in these applications.^95^ In a distinctive application, the MG-CS TENG showcases its transformative potential in repurposing COVID-19 clinical wastes. This TENG application not only addresses environmental concerns related to waste management but also harnesses the generated energy for smart sensing and various energy applications. By turning clinical waste into a resource for energy and sensing, MG-CS TENG represents an innovative solution with far-reaching implications in healthcare and environmental sustainability.^98^ Adding to the spectrum of applications, the SP-TENG takes on a dual role by acting as a capacitor. This TENG efficiently charges and powers various gadgets utilizing renewable energy sources. Its capacity to effectively store and deliver energy enhances its utility in diverse settings, emphasizing its role in promoting renewable energy practices and contributing to the development of self-powered systems. Together, these miscellaneous applications showcase the adaptability of TENGs in addressing unique challenges across different domains, spanning from electronic gadgetry to environmental sustainability.^62^

## 6. Potential opportunities and challenges in the implementation of waste-based TENGs

### 6.1. Electrical output performance limitations

Limitations in electrical output performance constitute essential constraints that dictate the efficiency, power density, voltage stability, and current delivery capabilities of energy conversion and power generation systems. These limitations stem from a complex interplay of thermodynamic principles, material properties, parasitic losses, and device architecture. Recent research across various energy harvesting and conversion technologies has identified consistent patterns in the manifestation of these constraints and potential strategies for their mitigation. Despite notable advancements and a substantial increase in output observed in various TENG devices since their inception, many still contend with suboptimal electrical output, particularly in the realm of low AC. Some authors posit that the diminished output from textile devices may be correlated with exceedingly low levels of contact area.^106,142,170–172^ In fact, recent studies demonstrated the correlation between TENG output and the extent of ‘real contact area’ was established. Consequently, the effective contact area experiences augmentation commensurate with the applied contact force. A challenge inherent in certain TENG applications, such as wearables, resides in the limited availability of normal contact force to bring the surfaces into sufficient proximity.^173–176^

An essential electrical limitation stems from the intrinsic material properties of waste-derived feedstocks. In contrast to engineered polymers such as fluorinated ethylene propylene (FEP) or polydimethylsiloxane (PDMS), which are specifically designed for high electron affinity and surface charge retention. Waste materials exhibit significant variability in their chemical composition, molecular weight distribution, and additive content. This heterogeneity directly influences their position in the triboelectric series and their ability to generate and maintain a stable surface charge. For instance, post-consumer PVC sourced from food packaging may contain plasticizers, stabilizers, and contaminants that exacerbate electrical leakage and diminish effective charge density, thereby constraining *V*_oc_ and *I*_sc_.^177^

Similarly, biowaste materials like chitosan and keratin, despite their sustainable benefits, often face challenges related to hygroscopicity, which can diminish dielectric strength and result in charge dissipation under typical conditions. However, recent advancements suggest that meticulous material engineering can mitigate these performance limitations. By integrating keratin derived from recycled fur with chitosan, a composite TENG was created, achieving a 375% increase in output voltage and an impressive power density of 14.4 W m^−2^, while also demonstrating remarkable stability over 8000 cycles.^178^ Additionally, compared to traditional TENG architectures, the employment of auxetic metamaterials structures with a negative Poisson's ratio has resulted in a four-fold increase in *V*_oc_ and this design idea is easily transferable to waste polymer matrices.^179^ TENGs still have intrinsic electrical limits despite material improvements. The majority of commercial electronics that require steady, low-voltage DC power are incompatible with their high-voltage (hundreds of volts), low-current (microamps), and pulsed AC outputs. Because of this, sophisticated power management circuits (PMCs) are required to control energy storage, voltage regulation, and rectification.^13^

Recent investigations by other scholars underscore the pivotal role of surface texturing, in addition to physio-chemical properties, influencing interfacial surface phenomena. Further research endeavors should prioritize the advancement of a more comprehensive comprehension of the specific response to texture at a local scale and the resultant collective response of the global system. This enhanced understanding would significantly contribute to the development of optimized surface textures and structures, thereby optimizing tribocharge density and augmenting the overall performance of TENGs. Notably, recent strides in *in situ* real contact visualization systems, as highlighted in reference, could offer invaluable assistance in systematically exploring this critical aspect. Various methodologies for surface and sub-surface modification have been deployed in the pursuit of enhancing TENG output.^180–182^ These include plasma treatments, chemical treatments, micro/nano-structuring of surface topography, and the utilization of ferroelectric materials with appropriate nano fillers, along with the introduction of low permittivity substrates.^183–187^

An additional noteworthy development involves the substantial augmentation of charge density by placing the TENG in a vacuum environment, thereby circumventing the air breakdown limit dictated by Paschen's law. This innovative approach led to the achievement of a remarkable record charge density of 1003 µC m^−2^. Several approaches aimed at enhancing TENG output, particularly those pertaining to material and topographical modifications, have demonstrated increased efficiency. However, in some instances, the improvements have occurred without a concomitant elucidation of the underlying mechanisms. As we approach a decade since the publication of the first TENG paper, substantial progress has been achieved. Nevertheless, further advancements are imperative to render TENG output more viable for practical applications. To achieve this, sustained efforts are required to delve into the fundamental physics of triboelectrification. While rapid progress has been made, a more comprehensive understanding of the underlying principles is essential. This enhanced understanding will serve as a guiding framework for the development of improved materials, surfaces, and designs, particularly for the next generation of TENGs. Consequently, a concerted focus on exploring the fundamental aspects of triboelectrification is pivotal to fostering the continued evolution and optimization of TENG technology.^188–191^

### 6.2. Fabrication and scalability challenges

The challenge of manufacturing poses a significant hurdle in the current landscape of TENGs. Presently, TENGs are manually fabricated for small-scale testing, necessitating substantial advancements to facilitate large-scale production. If new infrastructure were to be established, it must possess adaptability to keep pace with evolving designs and ongoing research efforts, as underscored in reference. This manufacturing challenge extends beyond the assembly of TENGs to the production of the materials themselves. Many materials employed in the construction of more efficient TENGs are intricate, involving multiple processing steps, thereby diminishing the cost efficiency of certain TENG solutions. Furthermore, marketing TENGs introduces an additional dimension to the challenge. Despite establishing themselves as a reliable green energy alternative, for TENGs to attain viability in the marketplace, they must be tailored for specific applications while maintaining a high level of cost efficiency. In a broader context, it is also prudent to explore advanced materials processing methods, particularly those that involve the repurpose of plastic material from waste, to be utilized in TENG devices. This multifaceted approach, encompassing manufacturing scalability, material complexity, and strategic design for market penetration, is imperative for overcoming the existing barriers and realizing the full potential of TENG technology.^192–195^

### 6.3. Environmental sustainability

The issue of robustness and durability of TENG devices has been acknowledged in existing literature. However, the majority of these studies have been conducted under controlled research conditions, with limited exploration into the robustness of TENG devices when exposed to extreme environmental conditions, ranging from elevated temperatures to machine washing of fabric-based TENGs. Material selection emerges as a pivotal factor in addressing the robustness challenge, as the choice of materials significantly influences the operational lifespan of a TENG device. While TENGs constructed from polymers such as PDMS and PTFE have theoretically demonstrated stability at temperatures ≥300 °C, the suitability of these polymer materials for specific environmental contexts remains a subject of consideration. In instances where the designated environment necessitates alternative materials, further research is imperative. Moreover, TENGs involve the repetitive contact or sliding of relatively thin layers of comparatively soft polymers. Extensive efforts are warranted to extend the wear and fatigue life of TENG devices, particularly for applications requiring prolonged energy generation. An additional concern pertains to the potential discrepancy between laboratory-recorded TENG output and its performance in real-world scenarios. Laboratory measurements are typically conducted under optimized conditions of surface contact, frequency, and amplitude. In practical working environments, TENGs may encounter challenges such as corrosion, wear, and unpredictable actuation forces, which can significantly impact output. Thus, a comprehensive investigation into the lifetime response of TENGs in realistic and dynamic environments is imperative, as highlighted in ref. 196 and 197.

### 6.4. Material standardization and quality control

The absence of a comprehensive method for measuring and comparing TENG output and efficiency remains a notable challenge. Although metrics such as *V*_oc_, *I*_sc_, and power density are commonly employed for comparative analysis, the reported values often involve only two of these parameters. Furthermore, the lack of standardization in experimental parameters, such as effective TENG size and applied pressure, introduces significant variability. The absence of a universally accepted method for TENG comparison hampers progress in the field. Attempts have been made to address this challenge, with Zou *et al.* proposing a method for quantifying the triboelectric series in 2019. However, this proposed method has yet to be widely accepted as a standardized approach.^198–201^ Despite these difficulties, TENGs are incredibly versatile, allowing for their use in a wide range of contexts. TENGs have already found use as power sources for electrocatalytic systems, micro-welding, self-powered microsystems, and medical applications. Future applications are expected to include their pivotal role in the evolution of the internet of things, particularly in domains like human–machine interface. Given the field's rapid development, TENGs have the potential to emerge as the fourth major form of green energy, along with solar, wind, and wave energy. TENGs have the potential to become one of the most versatile energy generators.^202,203^

## 7. Conclusion and future outlook

Waste-based triboelectric nanogenerators have emerged as a highly promising and sustainable technology for renewable energy harvesting, offering an environmentally friendly pathway for converting abundant waste materials into value-added functional devices. This review comprehensively discussed the diverse categories of waste-derived materials utilized in TENG fabrication, including agricultural residues, food waste, lignocellulosic biomass, textile waste, plastic waste, paper waste, and industrial by-products. Waste-derived materials, contingent upon their composition, can offer benefits including affordability, local accessibility, potential for resource recovery, recyclability, biodegradability for certain biogenic wastes, and advantageous surface chemistry, presenting a compelling alternative to traditional synthetic triboelectric materials while also tackling significant waste management and environmental pollution issues. The fundamental working mechanisms of triboelectric nanogenerators, including contact electrification and electrostatic induction, were examined alongside the major operational modes and material design strategies that govern device performance. Recent advancements demonstrate that appropriate surface modification, chemical treatment, nanostructuring, hybridization, and composite formation can significantly enhance charge generation efficiency, mechanical durability, and power output of W-TENGs. Furthermore, the integration of conductive fillers, biopolymers, and nanomaterials has enabled the development of flexible, lightweight, and wearable energy-harvesting systems suitable for next-generation self-powered electronics. The review also highlighted the broad spectrum of energy and sensing applications of W-TENGs, including biomechanical energy harvesting, wearable and portable electronics, environmental monitoring, smart textiles, healthcare sensors, Internet of Things (IoT) devices, and low-power energy storage systems. These applications illustrate the strong potential of waste-derived TENGs to contribute toward sustainable energy technologies and circular economy principles by simultaneously valorizing waste and generating clean energy.

Despite significant progress, several challenges still hinder the large-scale commercialization and practical implementation of W-TENGs. Issues related to material uniformity, long-term stability, moisture sensitivity, scalable fabrication, power management, and standardization of performance evaluation require further investigation. In addition, the relatively low and fluctuating electrical output under varying environmental conditions remains a key limitation for continuous real-world applications. Nonetheless, the practical advancement of waste-derived TENGs is limited by the intrinsic variability of waste feedstocks, inconsistencies between batches, and the decline in triboelectric efficiency after prolonged usage. To guaranty that waste based TENGs offer true sustainability benefits, future studies should thoroughly assess the trade-offs between pretreatment, chemical modification, durability, processing energy, and total environmental benefits. Future research should also focus on developing advanced waste-derived functional materials, improving device durability and efficiency, optimizing structural designs, and integrating intelligent energy storage and management systems. Waste-derived TENGs must progress from material evaluation and experimental demonstrations to mechanism-informed design, scalable production, and real-world industrial applications. Increased focus is necessary on comprehending the interconnections between waste composition, surface chemistry, triboelectric polarity, shape, and charge-transfer dynamics to facilitate informed material selection and alteration. To enable accurate comparisons of electrical performance between various waste-based TENGs, standardized testing procedures are also required. To verify their true sustainability benefits, long-term durability, environmental stability, processing costs, and life-cycle impacts should also be methodically assessed. Practical application may be further enhanced by integration with energy-storage devices, power-management circuits, and low-power electronics. Multifunctional waste-derived composites and data-driven methods for material discovery should be investigated in future research. In the end, a circular-resource framework that balances electrical performance, durability, scalability, economic viability, and environmental sustainability will be necessary for waste-based TENG development to be successful. However, the practical development of waste-based TENGs remains constrained by the inherent heterogeneity of waste feedstocks, batch-to-batch variations, and degradation of triboelectric performance during long-term operation. Future research should therefore critically evaluate the trade-offs between pretreatment, chemical modification, durability, processing energy, and overall environmental benefits to ensure that W-TENGs provide genuine sustainability advantages rather than simply shifting environmental burdens.

## Author contributions

Barshan Dev conceptualized the whole study, supervised, designed and writing of the manuscript, and critically reviewed the manuscript, Mitu Gharami was responsible for the collection of literature, data interpretation, and writing of this manuscript, Khadiza Newaz was responsible for writing of the manuscript and data collection, Md Ashikur Rahman performed the data collection, investigation, validation of this manuscript.

## Conflicts of interest

The authors have no relevant conflicts of interest to disclose.

## Data Availability

No primary research results, software or code have been included and no new data were generated or analysed as part of this review.
